# Pitfalls and opportunities for applying latent variables in single-cell eQTL analyses

**DOI:** 10.1186/s13059-023-02873-5

**Published:** 2023-02-23

**Authors:** Angli Xue, Seyhan Yazar, Drew Neavin, Joseph E. Powell

**Affiliations:** 1 Garvan-Weizmann Centre for Cellular Genomics, Garvan Institute of Medical Research, Sydney, NSW 2010 Australia; 2 School of Biomedical Sciences, University of New South Wales, Sydney, NSW 2052 Australia; 3 UNSW Cellular Genomics Futures Institute, University of New South Wales, Sydney, NSW 2052 Australia

**Keywords:** Single-cell RNA-seq, Pseudo-bulk, Latent variable, PEER factors, Principal component analysis, Normalization, eQTL mapping

## Abstract

Using latent variables in gene expression data can help correct unobserved confounders and increase statistical power for expression quantitative trait Loci (eQTL) detection. The probabilistic estimation of expression residuals (PEER) and principal component analysis (PCA) are widely used methods that can remove unwanted variation and improve eQTL discovery power in bulk RNA-seq analysis. However, their performance has not been evaluated extensively in single-cell eQTL analysis, especially for different cell types. Potential challenges arise due to the structure of single-cell RNA-seq data, including sparsity, skewness, and mean-variance relationship. Here, we show by a series of analyses that PEER and PCA require additional quality control and data transformation steps on the pseudo-bulk matrix to obtain valid latent variables; otherwise, it can result in highly correlated factors (Pearson's correlation r = 0.63 ~ 0.99). Incorporating valid PFs/PCs in the eQTL association model would identify 1.7 ~ 13.3% more eGenes. Sensitivity analysis showed that the pattern of change between the number of eGenes detected and fitted PFs/PCs varied significantly in different cell types. In addition, using highly variable genes to generate latent variables could achieve similar eGenes discovery power as using all genes but save considerable computational resources (~ 6.2-fold faster).

**Supplementary Information:**

The online version contains supplementary material available at 10.1186/s13059-023-02873-5.

## Introduction

Inferring latent variables that explain the variations in the gene expression data has been an essential step for expression quantitative trait locus (eQTL) analyses. It can be used to identify the unobserved confounding effects and potential cellular phenotypes (e.g., transcription factor or pathway activation). Popular methods include principal component analysis (PCA) [1], surrogate variable analysis (SVA) [2], and probabilistic estimation of expression residuals (PEER) [3, 4]. PCA is a well-established method for latent variable inference and has been implemented in eQTL analyses [5, 6]. PEER implements a Bayesian framework to estimate the latent variables and jointly learns the contribution to the gene expression variability from known covariates and hidden factors. The inferred factors (i.e., PEER factors) can be applied to increase the power of eQTL discovery. This method was introduced in 2010 and has been widely used in bulk eQTL analyses [7–10], and recently, the emerging field of single-cell RNA-sequencing (scRNA-seq) pseudo-bulk eQTL analysis [11–14].

As the scale of scRNA-seq studies rapidly grows, eQTL analyses that use pseudo-bulk approaches have emerged. Pseudo-bulk refers to the aggregation of the gene expression profiling of all cells from one sample into a single pseudo-sample; thus, the expression matrix dimension will be assimilated as the bulk RNA-seq data as a “sample x gene” matrix. However, due to the nature of scRNA-seq data structures, the bulk expression matrix and scRNA-seq pseudo-bulk expression matrix can be very different. There are three main differences: matrix sparsity, distribution normality or skewness, and mean–variance dependency. First, since the scRNA-seq matrix is sparse and many elements are zero, the pseudo-bulk gene expression matrix still contains many zeros. Second, some evidence showed that the underlying distribution of gene expression across cells largely follows non-normal distributions, such as Gamma, Point-Gamma, or non-parametric distributions [15], and inter-individual distributions of mean gene expression in pseudo-bulk matrix of many genes could be non-normal and heavily right-skewed. Third, mean–variance dependency exists between the intra-individual mean and variance due to the characteristics of the underlying distribution, and such relationships could be retained between the inter-individual mean and variance. These features mentioned above of pseudo-bulk data may violate the assumptions of the PEER method. Consequently, the inferred PEER factors (PFs) could suffer from biases or spurious correlations with each other, which might lead to the problematic interpretation of the factors themselves and compromise the discovery power of pseudo-bulk eQTL association.

Moreover, how many PFs/PCs should be fitted in the eQTL association model to improve the discovery power for pseudo-bulk data is unclear. Previous bulk eQTL analysis either chose a fixed number [9] or a certain threshold based on the sample size [7, 10]. Some studies have run sensitivity tests [7, 8, 10], but such optimization has not been systematically evaluated for single-cell data at the population-scale level. A recent study [16] has evaluated the automatic elbow detection method and Buja and Eyuboglu (BE) algorithm for PCA in bulk eQTL analysis. However, such an investigation has not been conducted for single-cell pseudo-bulk data. Another recent study [13] compared the performance between different inference methods for single-cell pseudo-bulk data but only in induced pluripotent stem cells (iPSCs).

Here, we identify some common scenarios where pitfalls occur when inferring latent variables in single-cell eQTL analyses and how they can be avoided with data-driven approaches. We have performed analyses using PEER factors and PCA, where we have observed spurious correlations among the inferred factors. To help with the future application of PEER and PCA to single-cell RNA-seq data, we propose guidelines for the quality control and scaling of the pseudo-bulk expression matrix, diagnosing and troubleshooting the inferred latent variables, and a new way to select the optimal number of latent variables to improve the eQTL discovery.

## Results

### Behavior of latent variants under different quality control and transformation options

**Fig. 1:**
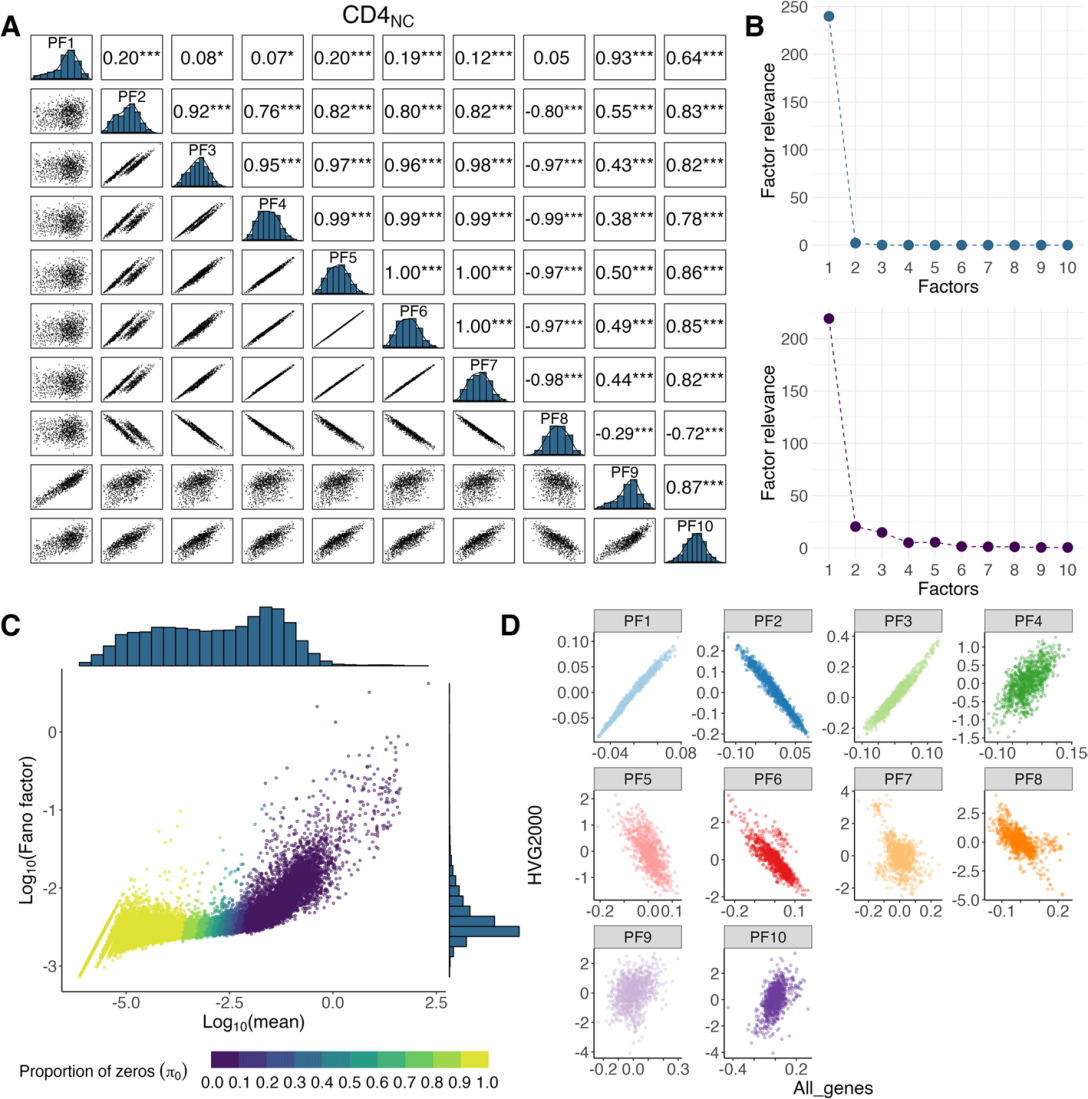
Correlation among inferred PEER factors and global intra-individual mean–variance dependence. **A** Pair-wise correlation plot among the first 10 PEER factors generated from single-cell expression in CD4_NC_ (Naive CD4) cells without any quality control (option #1). The upper triangle panel shows the pair-wise estimates of Pearson’s correlation, and the bottom triangle panel shows the pair-wise scatter plot between the PEER factors. The diagonal panel shows the distribution of each PEER factor. The significance of the correlation test is annotated by * *p*-value ≤ 0.05, ** ≤ 0.01, *** ≤ 0.001. **B** Diagnostic plot of the factor weights without further quality control on the pseudo-bulk matrix (option #1, upper panel) and option #11 QC (lower panel). **C** Relationship between intra-individual pseudo-bulk mean and Fano factor per gene. Both axes are Log10 transformed. The color of the dots indicates the proportion of zero expression across individuals (${\pi }_{0}$) for each gene. **D** Scatter plot of first 10 PEER factors generated from all genes against those from top 2000 highly variable genes (option #11 vs option #12)

We investigated how PEER factors behave under different quality control (QC) and transformation options on the pseudo-bulk matrix using three independent scRNA-seq datasets: one from peripheral blood mononuclear cells (PBMCs, *N* = 980) and the others from fibroblast cells and iPSCs [12] (*N* = 79 and 31). To construct a pseudo-bulk expression matrix for each cell type, the gene expression level per individual was calculated as the intra-individual mean counts across cells (see the “Methods” section). We first generated PFs while including sex, age, and six genotype PCs as covariates. We observed strong correlations among PFs (see CD4_NC_ cells as an example in Fig. 1, and other cell types shown in Additional file 1: Fig. S1). For instance, while most known covariates are not correlated (Pearson’s *r* =  − 0.04 ~ 0.06, except − 0.13 between PC3 and PC4; Additional file 1: Fig. S2), the first and second PFs show a modest correlation (Pearson’s *r* = 0.20) and the correlations among PFs 5–7 are equal to 1. Although the hidden factor model of PEER allows for non-orthogonal components, the mean of the pair-wise Pearson’s *r* across the first 10 PFs were all larger than 0.5 in all 14 cell types, suggesting that many PFs are redundant and overfitted. Additionally, we found that the variance explained by the first PF was overwhelmingly larger than the rest of the PFs, where the latter’s contributions seem negligible (upper panel in Fig. 1B and Additional file 1: Fig. S1B). Another issue is that, due to the sparsity in scRNA-seq data, there is a certain proportion of genes whose intra-individual expression is mostly zero (Fig. 1C); therefore, regardless of what the transformation or normalization methods are used, the intra-individual distribution of these genes will still be strongly right-skewed which violates the normality assumption of PEER (see examples in Additional file 1: Fig. S3).

**Fig. 2:**
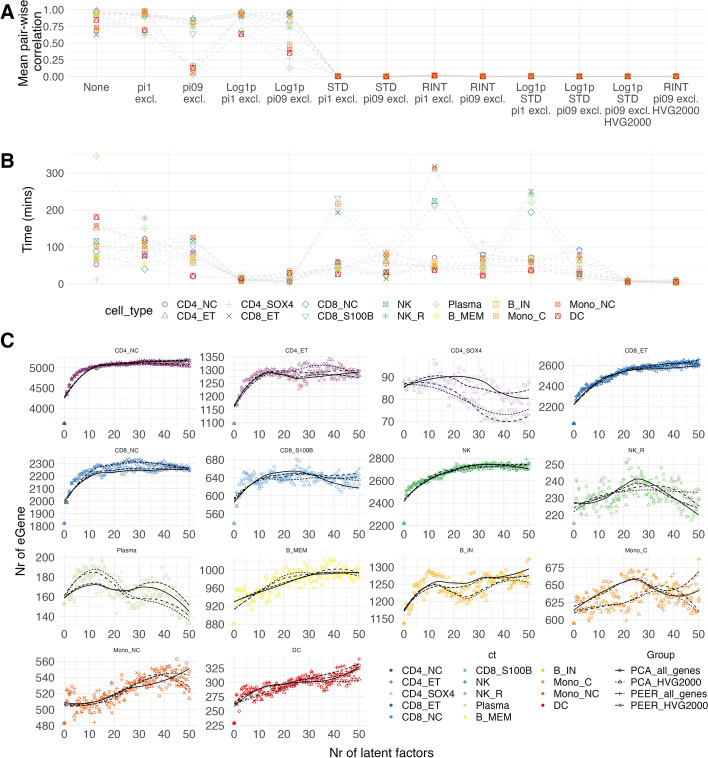
Performance of different QC options on generation of PEER factors and sensitivity test for eGene detection using PEER and PCA. **A** The mean pair-wise correlation among the first 50 PEER factors. Each color and shape represent a specific cell type. **B** Time to generate 50 PEER factors by different quality control options on the pseudo-bulk matrix. **C** The *x*-axis denotes the number of PFs/PCs fitted as covariates in the association model. The *y*-axis represents the number of eGenes with at least one eQTL at local FDR < 0.05. The shape of each scatter point indicates whether generating PFs/PCs using all genes or the top 2000 highly variable genes (selected based on variance-to-mean ratio before transformation and scaling) to generate PEER factors (both excluded genes with ${\pi }_{0}\ge 0.9$, log(*x* + 1) transformed and standardized). The local regression lines are fitted for number of eGenes for number of latent variables. Different line types indicate four different scenarios

To alleviate the impact of these properties, we mixed and matched different options in combinations (13 options in total) to generate PEER factors: (1) excluding the genes with zero expression in more than a certain % across the individuals (i.e., ${\pi }_{0}\ge 0.9\mathrm{\ or\ }1$); (2) log(*x* + 1) transformation; (3) standardization, which scales the distribution to mean = 0 and standard deviation = 1; (4) rank-based inverse normal transformation (RINT); (5) selecting the top 2000 highly variable genes (HVGs, ranked by variance-to-mean ratio before the transformation and scaling) to generate the PFs (see the “Methods” section). The results showed that the correlations among PFs were still high even when genes with high ${\pi }_{0}$ were excluded and/or log(*x* + 1) transformed (options #1–5, Fig. 2A). Among options #6–11, option #7 (standardization + ${\pi }_{0}\ge 0.9$ excluded) and option #11 (log(*x* + 1) + standardization + ${\pi }_{0}\ge 0.9$ excluded) had the lowest mean pair-wise correlation among PFs (Fig. 2A and Additional file 1: Fig. S4). Between these two, we identified option #11 as optimal because the skewness of gene expression across individuals (measured by the Pearson’s moment coefficient of skewness, ${\widetilde{\mu }}_{3}$) was lower than option #7 (median skewness for all genes is 0.86 ~ 3.8 vs 0.90 ~ 5.12 across 14 cell types). We also tried to generate PFs using the top 2000 HVGs (options #12–13 in Fig. 2A, B), and they were highly correlated with those generated using all the genes (Fig. 1D and Additional file 1: Fig. S1D), highlighting that the HVGs can explain most of the variation that was explained when using all the genes and reduce the runtime from 46.2 min to 7.4 min on average for different cell types (Fig. 2B).

### Impact of latent variables on eQTL detection power

Next, we investigated how PEER factors generated from different options affect the eQTL discovery power. We calculated PFs using all genes or the top 2000 HVGs (both pre-excluded genes with ${\pi }_{0}\ge 0.9$) and compared the number of eGenes (at least associated with one significant eQTL) identified when incrementally fitting PFs as covariates from 0 to 50. Notably, the pattern of change in the number of eGenes varied across different cell types (Fig. 2). For CD4_NC_ cells, the number of eGenes continually increased until reaching an asymptote of around 30, while CD4_SOX4_ cells peaked between 10 and 15 and decreased as more factors were included. Also, the pattern of change in eGene discovery power was consistent regardless of using all genes or the top 2000 HVGs (Fig. 2C). These consistencies reaffirmed that using the top 2000 HVGs captures most of the latent variation that all genes can explain in this dataset. We also compared the number of eQTLs/eGenes when PFs were generated without QCs or by QC option #11. The latter can identify 9.0 ~ 23.1% more eQTLs or 1.7 ~ 13.3% more eGenes at the peak (Additional file 1: Fig. S5). It was also observed that the number of eGenes started to drop much earlier when incorporating highly correlated PFs (Additional file 1: Fig. S5). Performing these sensitivity analyses in new studies is time-consuming and computationally expensive, especially for large cohorts with many cell types. Our results showed that using the top 2000 HVGs to generate PFs could achieve similar power in eGene discovery compared to using all genes (Additional file 1: Table S1) while saving significant computational resources (~ 6.2-fold faster on average, Fig. 2B). The computational time for eQTL analyses given a different number of PFs was recorded in Additional file 1: Fig. S6.

Furthermore, the optimal number of fitted PEER factors is not solely dependent on sample size but on how much variation can be explained. For CD4_SOX4_ cells, the inferred PFs did not significantly increase the eGene detection power in most scenarios (Fig. 2C and Additional file 1: Table S1); therefore, selecting the number of PFs in eQTL association just based on sample size could be erroneous. To balance the discovery power and potential false positives, there are different methods to determine the optimal number of factors. Two commonly used methods are the automatic elbow detection method and Buja and Eyuboglu (BE) algorithm, which has been comprehensively evaluated for PCA in bulk RNA data [16]. We ran these two methods for PCs inferred from the single-cell data for each QC option (see the “Methods” section). The results showed that the BE algorithm selected an unexpectedly large number of PCs (mostly from 100 ~ 200), while the automatic elbow detection method mostly selected 3 ~ 22 (Additional file 1: Fig. S7). We speculate that this is because, in the single-cell data, the first few PCs explain the most variation. In the BE algorithm, randomly selecting *K* number of PCs will likely choose the PCs explaining a tiny proportion of the total variance. Accordingly, when comparing the variance explained by the first *K* PCs and randomly selected *K* PCs, the former is often much larger. These two methods are not directly applicable to PEER because the relevance factor is conceptually different from the variance explained for each PC. To overcome this, we proposed a “local greedy” algorithm to choose the optimal number of PFs, which takes the eQTL sensitivity results into account (see the “Methods” section). By this strategy, most cell types will be only adjusted with 2 ~ 10 PFs rather than 20 ~ 50 but retain ~ 71% power gain of eGene discovery (Additional file 1: Fig. S8). We further compare the elbow detection method and the local greedy algorithm for PCA (Additional file 1: Fig. S9). The elbow detection method identified, on average 0.6% fewer eGenes than the local greedy algorithm, but the optimal number of PCs was much higher (31 vs 7) across 14 cell types. Notably, in CD4_SOX4_ cells, the elbow detection method identified 20.5% fewer eGenes than the local greedy algorithm, which shows its disadvantage in single-cell pseudo-bulk eQTL analysis that it did not take eQTL sensitivity results into account.

### Effect of sample size on single-cell eQTL latent variables

To expand our exploration into other cell types, we tested the data from Neavin et al. [12], who noted that the number of detected eGenes dropped with the incremental increase of PFs in the four iPSC clusters but not in the six fibroblast clusters (*Figure S20* in the original paper). Strong correlations among PFs were also observed in four iPSC subtypes (after the 4th or 5th PF) but not in fibroblast subtypes (Additional file 1: Fig. S10). In the case of iPSC subtypes, fitting more PFs in the eQTL association analysis added more noise, which led to the loss of power. We hypothesize that the difference is due to the sample size since the input pseudo-bulk expression matrices were already quality-controlled using quantile normalization and *z*-transformation. There are rules of thumb for the minimum sample size required for factor analysis [17, 18], which suggest 3–20 samples per factor. When the sample size is small, the first few PFs explain almost all the variation, leaving little for the additional factors to explain. Thus, the following factors become strongly correlated due to overfitting (observed as similar or equivalent weights for certain PFs, Additional file 1: Fig. S1B). The sample sizes were 79 for fibroblast and 31 for iPSCs; thus, iPSCs are more likely to suffer from sample size bias. We validated our hypothesis by down-sampling the fibroblast dataset (*N* = 31 to match the iPSCs; see the “Methods” section). The mean of pair-wise correlations among 10 PFs ranged from 0.11 to 0.99 in the six fibroblast subtypes (Additional file 1: Fig. S11), indicating that insufficient sample sizes could result in high correlations among PFs even if the expression matrices were well normalized. We also down-sampled the fibroblast clusters to 40 and 50 separately and found a negligible correlation among inferred PFs when *N* = 50 but moderate correlations (0.004–0.390) when *N* = 40, suggesting that we might need at least five samples per factor in such a dataset.

As principal components (PCs) are also commonly used to control for confounding factors in the eQTL analysis, we also conducted the same exploration for PCs in the OneK1K cohort. The PCs inferred without proper QC and scaling also showed spurious correlations but were very modest compared to PFs (Additional file 1: Fig. S12). The eQTL sensitivity analyses showed that the number of eGenes detected was consistent with that using PEER factors. Similarly, the trend curves of the incremental number of latent variables overlap (Fig. 2 and Additional file 1: Fig. S13). These findings suggest that either PCs or PFs for single-cell eQTL mapping can be used to improve the number of eGene discoveries. However, the computational burden and flexibility are different between these two methods [13, 16].

## Discussion

Our results demonstrate that generating PEER factors and principal components requires more careful consideration in single-cell data. We recommend always checking the correlation among inferred latent variables (also with the known covariates) in single-cell pseudo-bulk data and conducting sensitivity analysis to select the optimal number of latent variables to be incorporated in eQTL mapping for each cell type. As we are moving towards the era of identifying single-cell, context-dependent, and dynamic eQTL [19–21], learning latent variables directly from single-cell level data [22, 23] and comparing them with those from pseudo-bulk would provide insights into the genetic control of gene expression at a more refined resolution.

Applying methods designed for bulk RNA-seq data to scRNA-seq pseudo-bulk data could be challenging as the assumptions might not be fully satisfied. This work highlights the pitfalls when learning PEER factors and principal components from scRNA-seq data. It presents diagnostic guidelines for performing further QC and normalization on single-cell data matrices to avoid spurious correlations among the inferred factors. Optimization for the number of latent factors included in the eQTL association model should be carried out by a data-driven approach. Using highly variable genes to generate the latent factors could achieve similar eGene discovery power as using all genes.

## Methods

Three single-cell datasets were used in this study to explore the performance of the PEER and PCA methods. The OneK1K consortium [14] is a population-scale single-cell RNA-seq dataset collected in Tasmania, Australia. This cohort includes 982 individuals, each with gene expression profiling for ~ 1000 (mean = 1297.0, standard deviation = 23.6) peripheral blood mononuclear cells (PBMCs). This dataset was quality controlled (QC), normalized and variance stabilized at the single-cell level by *sctransform* [24], and classified into 14 cell types by *scPred* [25] (see more details in ref [14]). We further identified two individuals with problematic metrics during the preliminary test of PEER (one with a deficient number of cells and the other with abnormal cell composition). We removed them in the primary analysis, ending up with 980 individuals. The final sample sizes for 14 different cell types range from 795 to 980 (Additional file 1: Table S1). Neavin et al. [12] collected 64,018 fibroblasts from 79 donors and 19,967 iPSC from 31 donors. The fibroblast data were classified into six subtypes, and iPSCs into four subtypes. For each subpopulation, the pseudo-bulk was calculated as the mean expression per gene per individual and then quantile-normalized and *z*-transformed.

PEER factors are latent variables that can explain the variability in gene expression. The original method [3] was proposed in 2010, and the software [4] was released in 2012. We used the R package “peer” (v1.0) to generate the PFs for the single-cell data applying max iterations = 2000 and the number of PFs = 50. Rank-based inverse normal transformation (RINT) was applied to the data by the function *RankNorm()* in the R package “RNOmni” [26]. The transformed matrix was standardized to a mean of zero with a unit standard deviation per gene. For analysis using the top 2000 HVGs, a refined gene list (pre-excluded genes with ${\pi }_{0}$ > 0.9 or mean < 0.001) was ranked by their variance-to-mean ratio (also known as Fano factor) before transformation and scaling. Note that these HVGs are not the same HVGs usually defined in the QC step of the raw expression matrix for single-cell data. The former indicates the genes with high mean variability across individuals, while the latter shows the genes that are highly variable across cells. We have varied different QC and transformation strategies to process the pseudo-bulk matrix to generate the PFs. There are 13 options in total:

**Table:** 

Option #	${\pi }_{0}$	Log1p	STD	RINT	HVG2000
1	= 1				
2	= 1				
3	≥ 0.9				
4	= 1	x			
5	≥ 0.9	x			
6	= 1		x		
7	≥ 0.9		x		
8	= 1			x	
9	≥ 0.9			x	
10	= 1	x	x		
11	≥ 0.9	x	x		
12	= 1				x
13	≥ 0.9				x

The ${\pi }_{0}$ indicates the threshold to filter out genes with high proportion of zero expression across individual. Log1p indicates log(*x* + 1) transformation per gene. STD indicates *z*-score scaling per gene. RINT indicates rank-based inverse normal transformation per gene. HVG2000 indicates whether to use top 2000 highly variable genes or all genes.

The eQTL association analysis was performed by Matrix eQTL (v2.3) [27]. We fit sex, age, the first six genotype PCs, and PEER factors as the covariates. We only tested the SNPs located in the *cis*-region of the gene within the 1 Mb from either upstream or downstream and with minor allele frequency > 5%. A local false discovery rate (LFDR) was calculated to control the false-positive rate for each chromosome tested by the R package “qvalue” [28]. An eGene was reported when at least one significant eQTL was found at LFDR < 0.05. The PEER factors were also generated without known covariates. The spurious correlations were also identified among these PFs, and no major difference in the eGene detection power was observed under both scenarios, whether using all genes or HVG2000.

To choose an optimal number of latent variables fitted in the eQTL association model, we propose a local greedy detection algorithm. We first calculated the percentage change of eGenes with every incremental latent variable added. Then, we performed a LOESS (locally estimated scatterplot smoothing) of the percentage change against the number of latent variables. Instead of choosing the number of PFs/PCs maximizing the number of eGenes, we selected the number of PFs/PCs right before the LOESS curve became negative. The rationale behind this algorithm is that if the number of eGenes reaches saturation, the percentage change is expected to be symmetrically distributed around 0. By this balanced strategy, we only need to adjust 2 ~ 10 PFs rather than 20 ~ 50 but can retain the most power gain of eGene discovery (Additional file 1: Fig. S8). The PFs and PCs are generated with HVG2000 using QC option #12 as an example when testing the algorithm. We also implemented the automatic elbow detection method and Buja and Eyuboglu (BE) algorithm [16] to identify the optimal number of PCs for all the QC options as a comparison. The parameters used in BE algorithm are as default (B = 20, alpha = 0.05).

### Comparison of performance between PCA and PEER

To investigate whether the strong correlation of PEER factors in iPSC data from Neavin et al. [12] arose due to the small sample size, we randomly down-sampled the six fibroblast subtypes from 79 to 31 individuals (to match the sample size of the iPSCs) 30 times and then generated PEER factors with these sub-samples. For each sub-sample, pair-wise Pearson’s correlations among 10 PEER factors were estimated. A similar down-sampling analysis was also conducted for sample sizes equal to 40 and 50.

## Supplementary Information

**Additional file 1: Fig. S1.** Correlation among inferred PEER factors and global intra-individual mean–variance dependence. **Fig. S2.** Correlation among known covariates, including sex, age, and first six genotype PCs. **Fig. S3.** Different transformations of highly expressed and lowly expressed genes. **Fig. S4.** Performance of the 8 candidate QC options of input matrix for PEER factor generation. **Fig. S5.** Sensitivity test for eQTLs and eGenes discovery power between no QC and QC option #11. **Fig. S6.** The computational time for eQTL association analysis when adjusting a different number of PEER factors. **Fig. S7.** Detection of the optimal number of PCs. **Fig. S8.** Detection of the optimal number of PEER factors using the local greedy algorithm. **Fig. S9.** Detection of the optimal number of PCs using elbow detection method and the local greedy algorithm. **Fig. S10.** The correlation plot of fibroblast and iPSC clusters from Drew et al. **Fig. S11.** Distribution of mean pair-wise correlation coefficient among PEER factors in down-sampling of fibroblast clusters. **Fig. S12.** Performance of different QC options on the generation of expression PCs. **Fig. S13.** Sensitivity test for eGene detection using a different number of latent variables inferred from PCA and PEER. **Table S1.** The maximum of eGene detection power gain by incorporating PEER factors.

**Additional file 2. **Review history.

## Data Availability

For the OneK1K dataset, the single-cell gene expression and genotype data are available via Gene Expression Omnibus (GSE196830) [29]. The cell by gene data is available at Human Cell Atlas (HCA) (https://cellxgene.cziscience.com/collections/dde06e0f-ab3b-46be-96a2-a8082383c4a1) [30]. Please note that cellxgene does not accept hg19 data so this one is based on hg38. For the fibroblast/iPSC datasets, the scRNA-seq data for all 79 fibroblast cell lines and 31 iPSC cell lines are available from ArrayExpress (Accession Number: E-MTAB-10060) [31]. The analysis code is available on Github [32] (https://github.com/powellgenomicslab/PEER_factors, under GPL-3.0 license) and Zenodo [33] (10.5281/zenodo.7513270, under Creative Commons Attribution 4.0 International license).
