# Supplementary material for: Pitfalls and opportunities for applying latent variables in single-cell eQTL analyses
Source: Genome Biol. 2023 Feb 23;24:33. doi: 10.1186/s13059-023-02873-5 (PMC9948363; doi:10.1186/s13059-023-02873-5)
Supplement: Supplementary file 1 — Additional file 1: Fig. S1. Correlation among inferred PEER factors and global intra-individual mean–variance dependence. Fig. S2. Correlation among known covariates, including sex, age, and first six genotype PCs. Fig. S3. Different transformations of highly expressed and lowly expressed genes. Fig. S4. Performance of the 8 candidate QC options of input matrix for PEER factor generation. Fig. S5. Sensitivity test for eQTLs and eGenes discovery power between no QC and QC option #11. Fig. S6. The computational time for eQTL association analysis when adjusting a different number of PEER factors. Fig. S7. Detection of the optimal number of PCs. Fig. S8. Detection of the optimal number of PEER factors using the local greedy algorithm. Fig. S9. Detection of the optimal number of PCs using elbow detection method and the local greedy algorithm. Fig. S10. The correlation plot of fibroblast and iPSC clusters from Drew et al. Fig. S11. Distribution of mean pair-wise correlation coefficient among PEER factors in down-sampling of fibroblast clusters. Fig. S12. Performance of different QC options on the generation of expression PCs. Fig. S13. Sensitivity test for eGene detection using a different number of latent variables inferred from PCA and PEER. Table S1. The maximum of eGene detection power gain by incorporating PEER factors. [file 13059_2023_2873_MOESM1_ESM.docx]

**Supplementary Figures
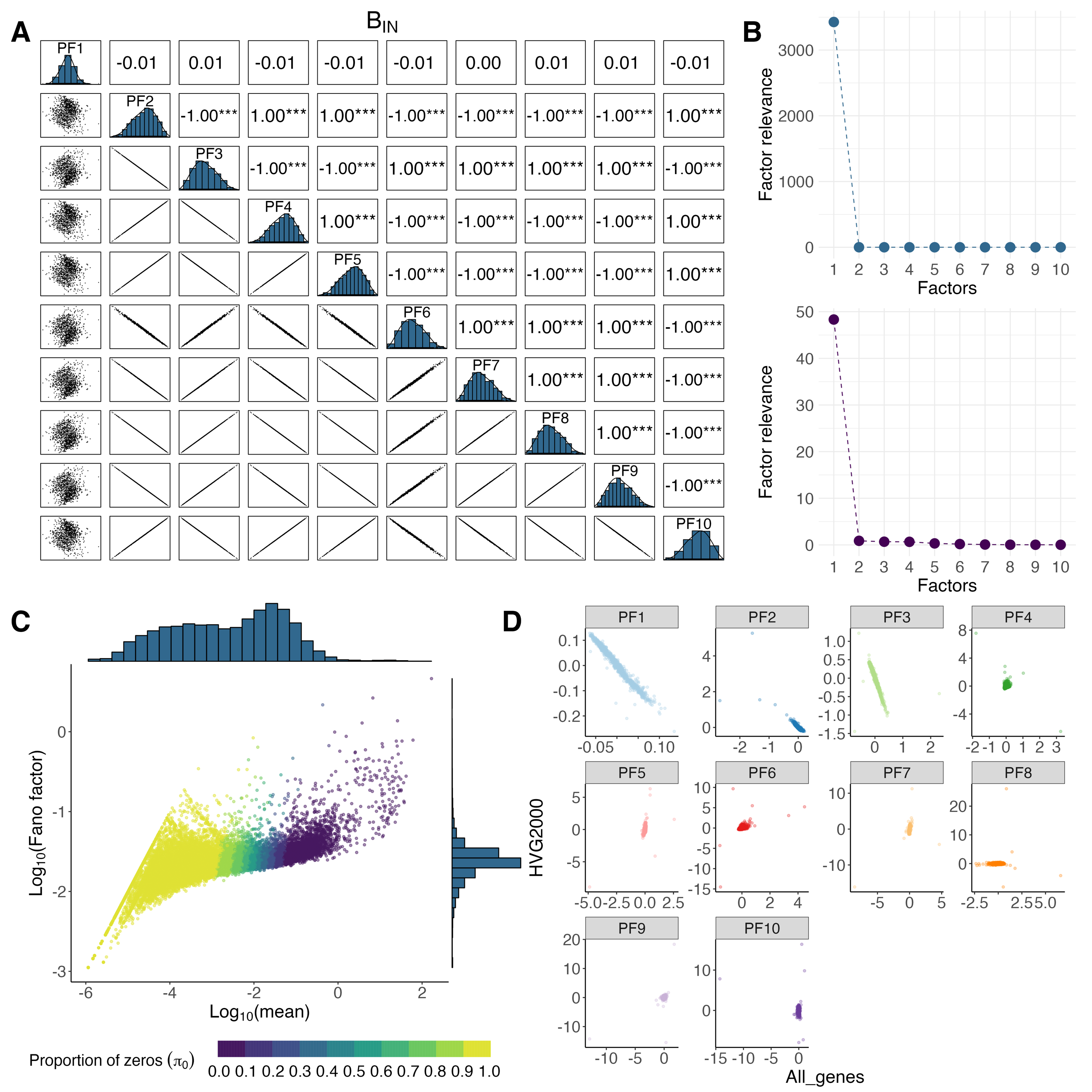

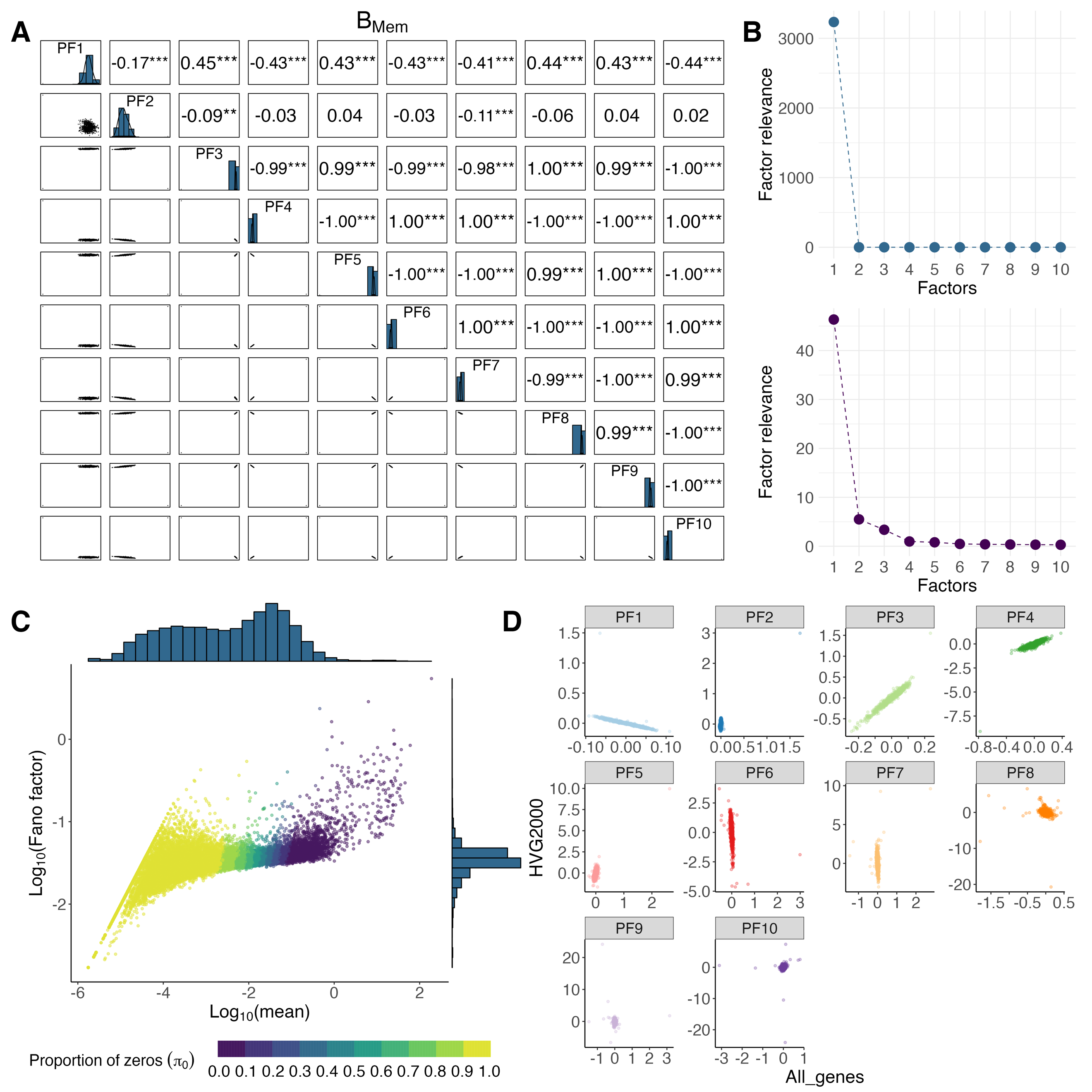

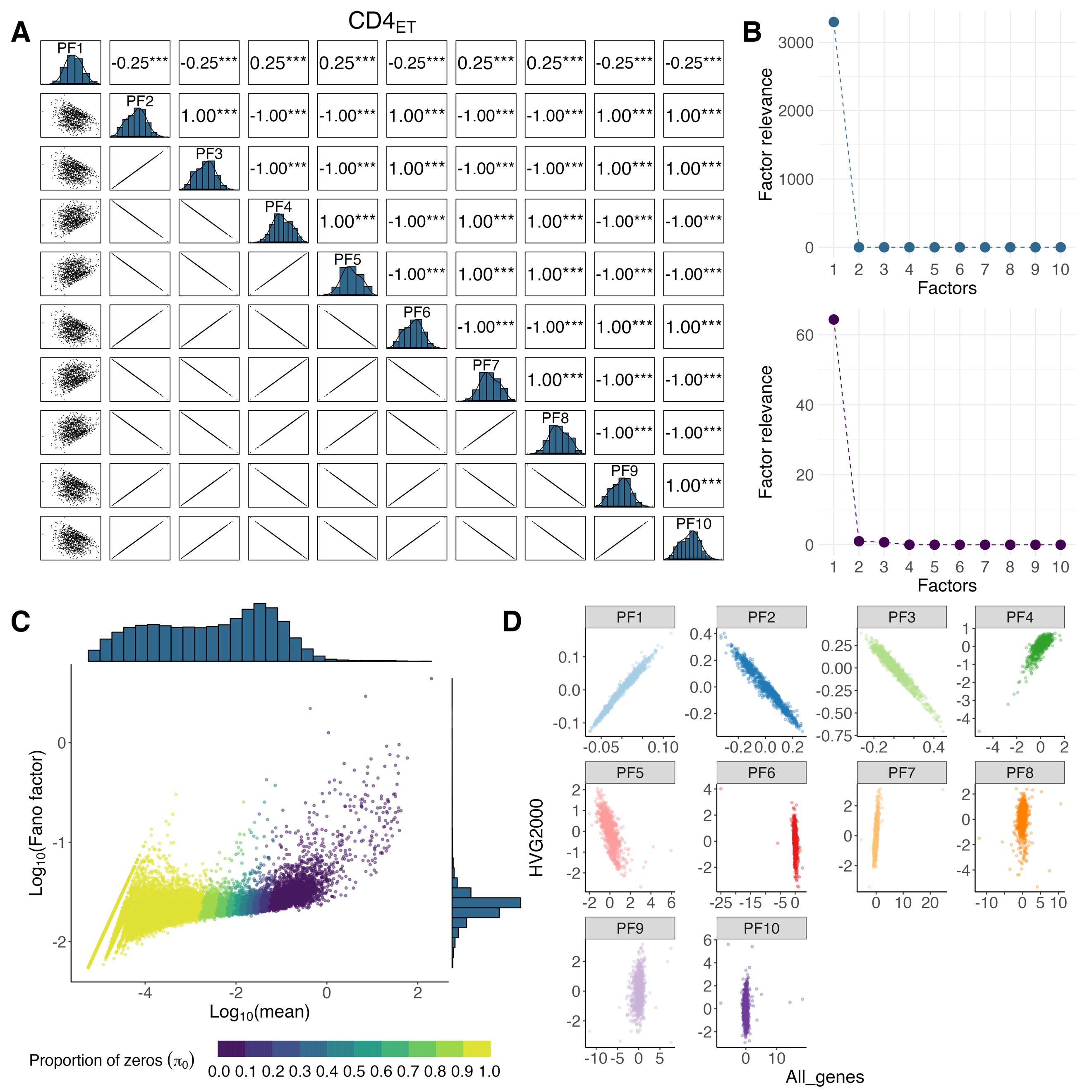

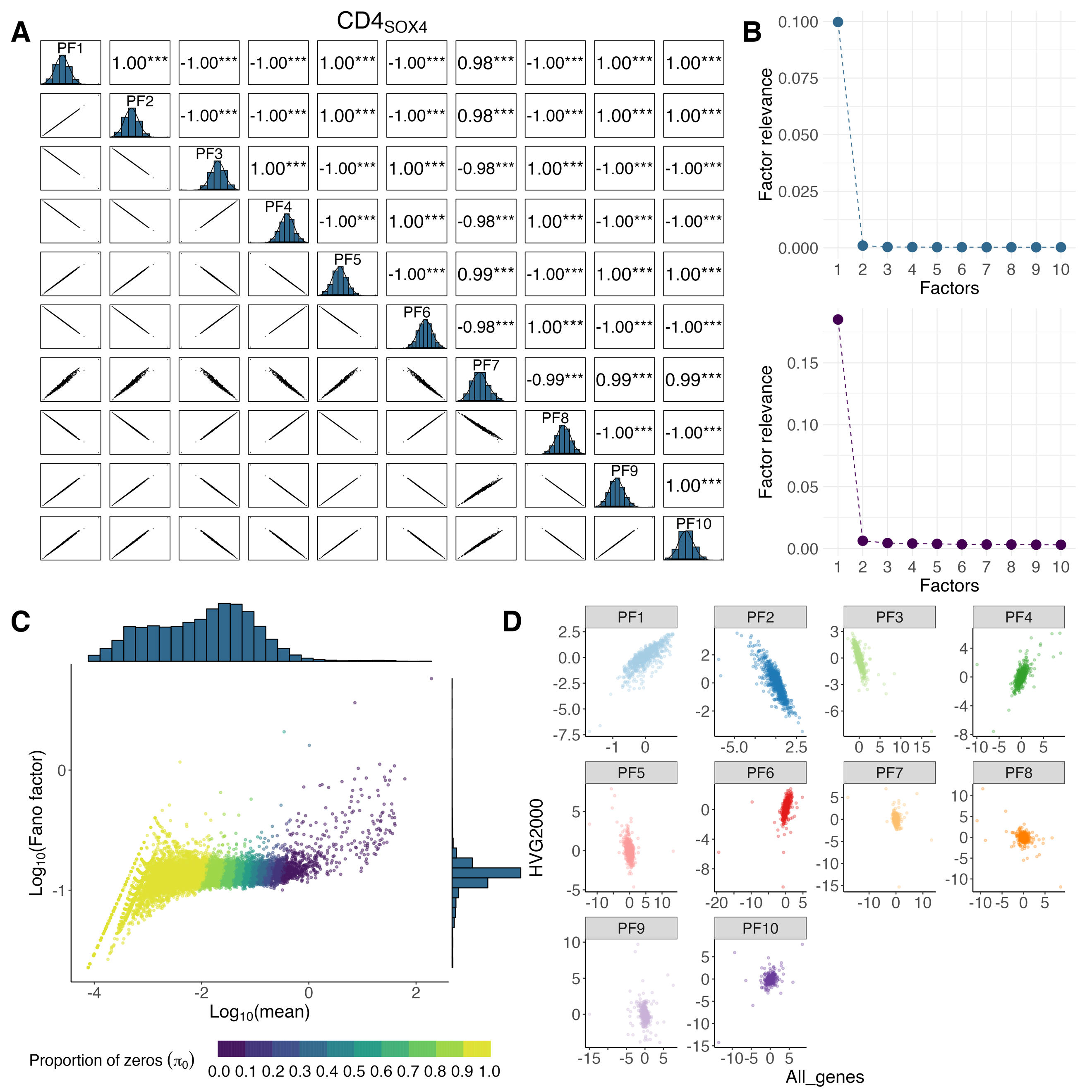

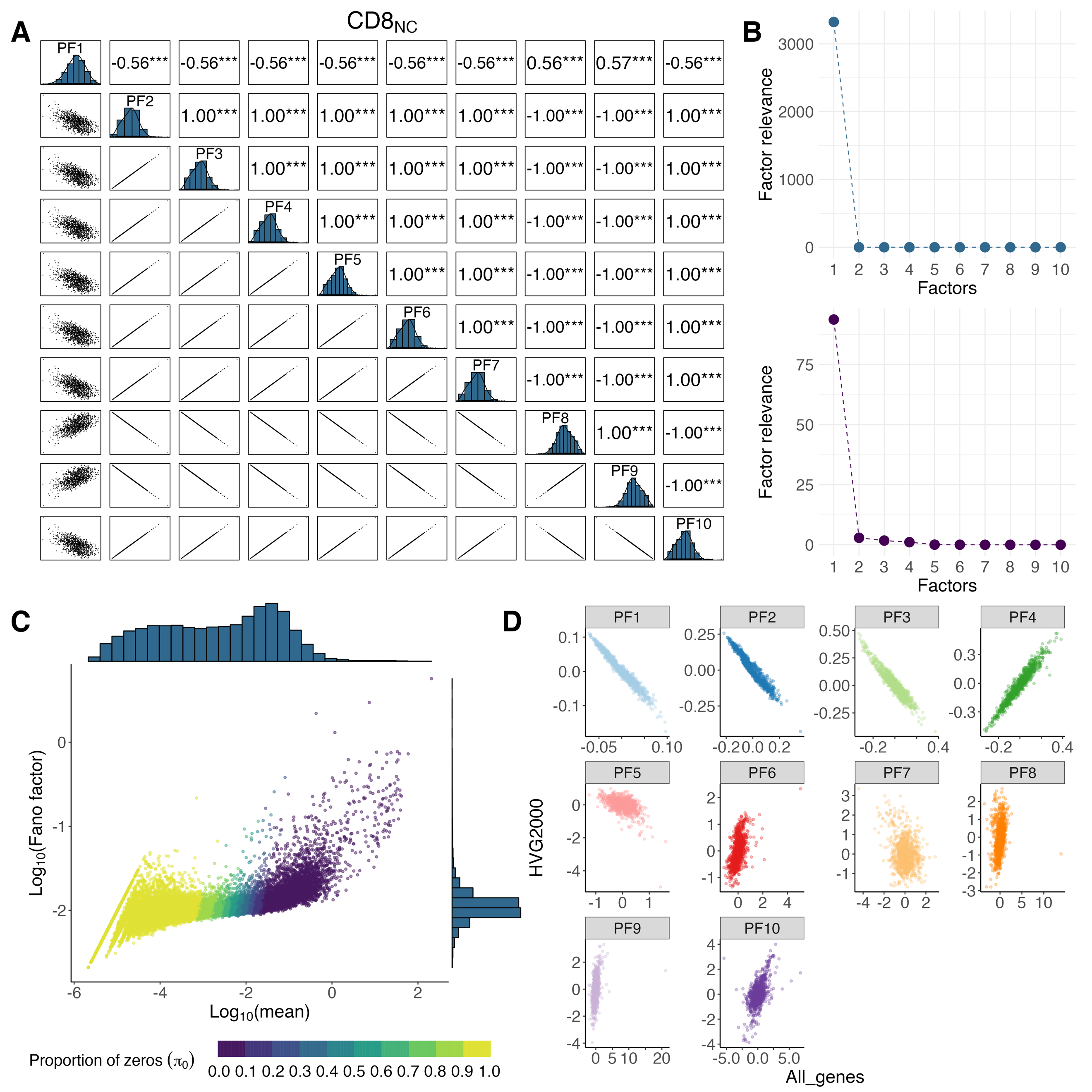

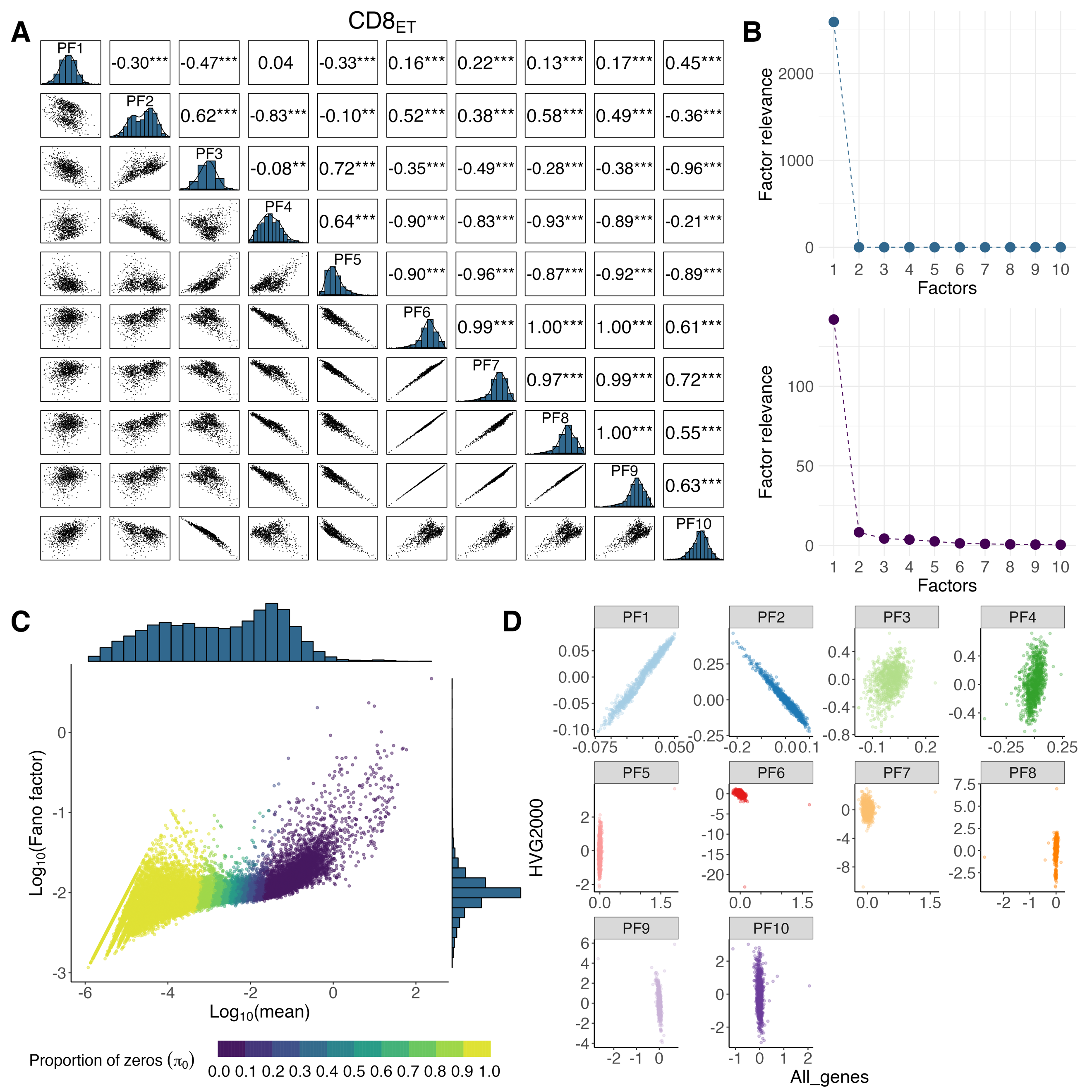

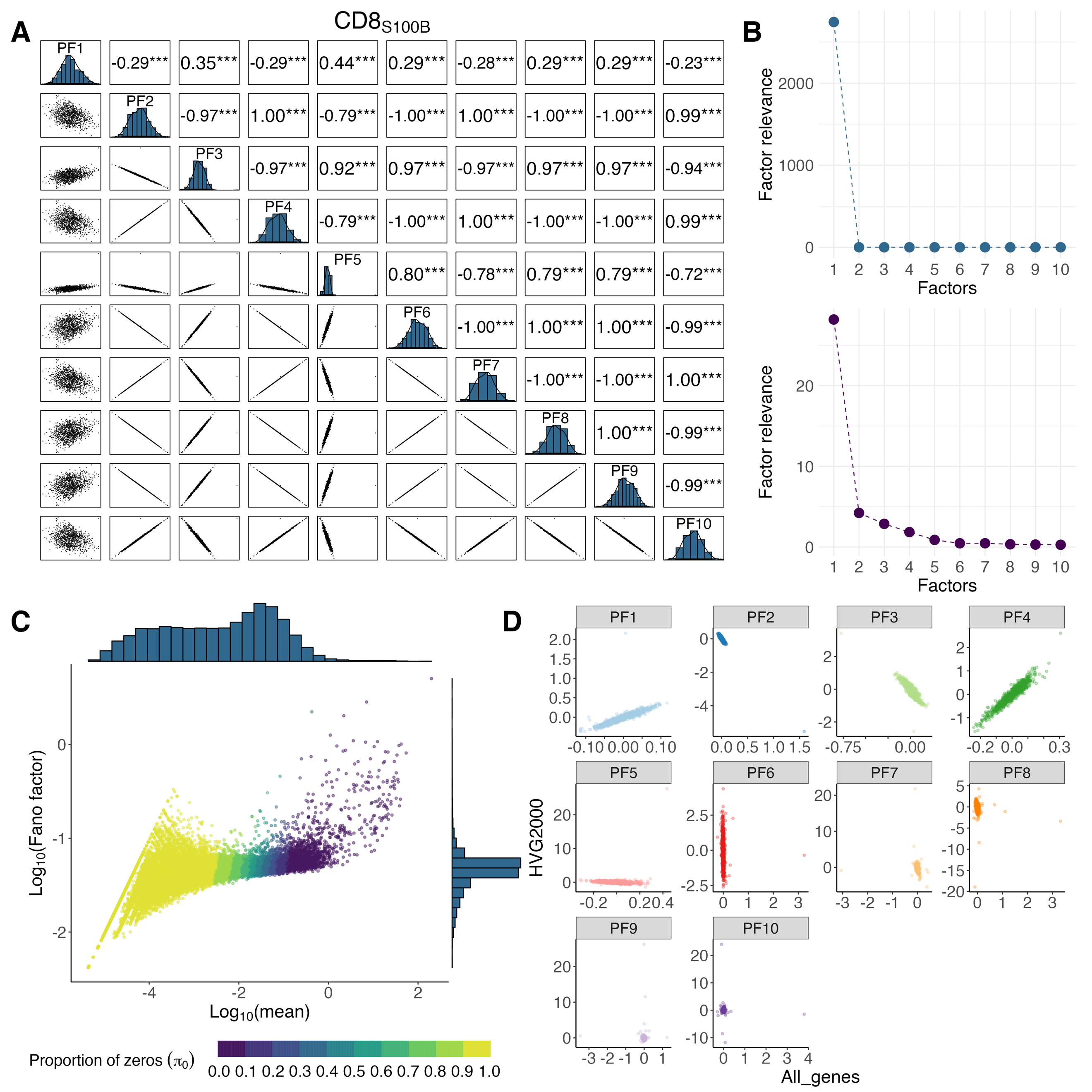

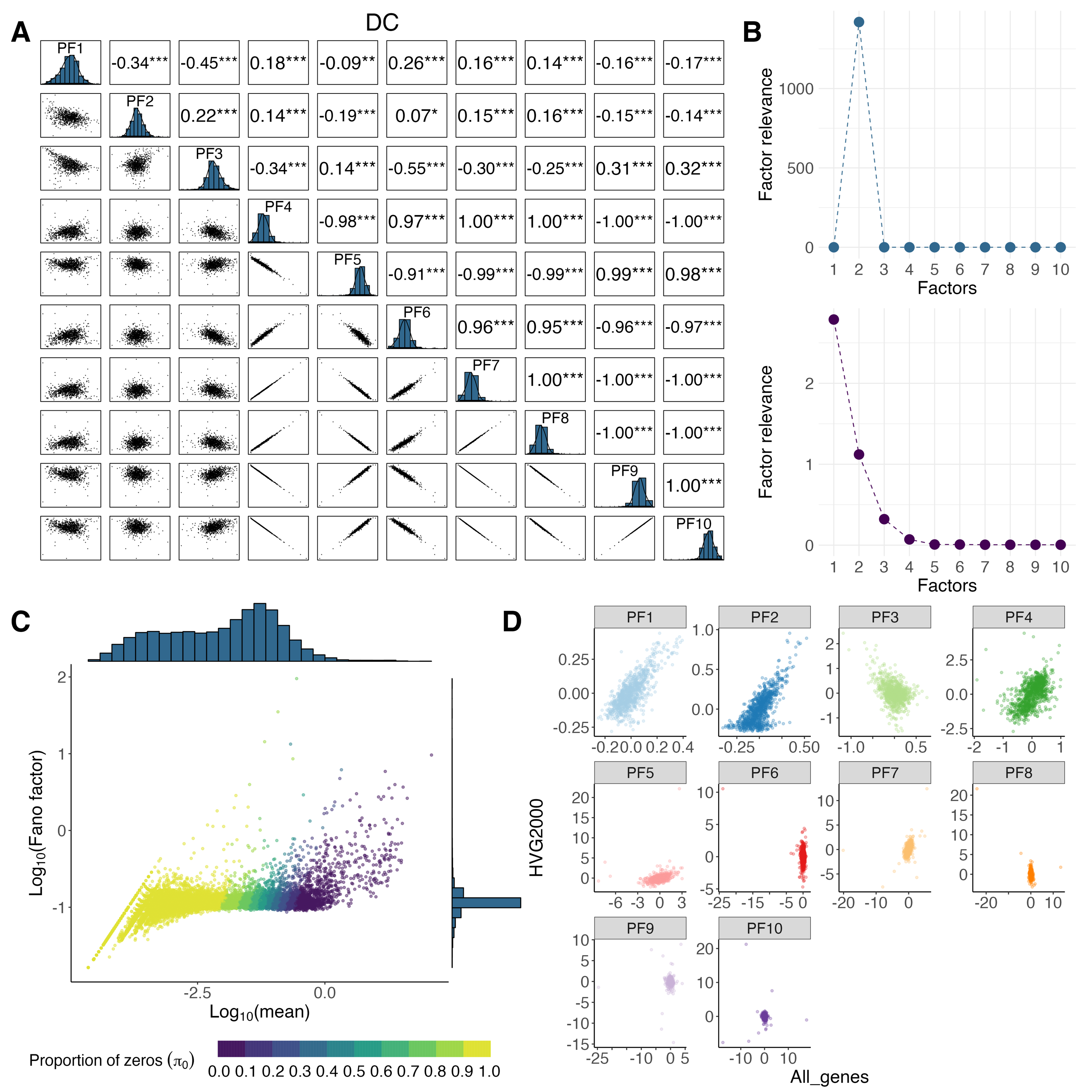

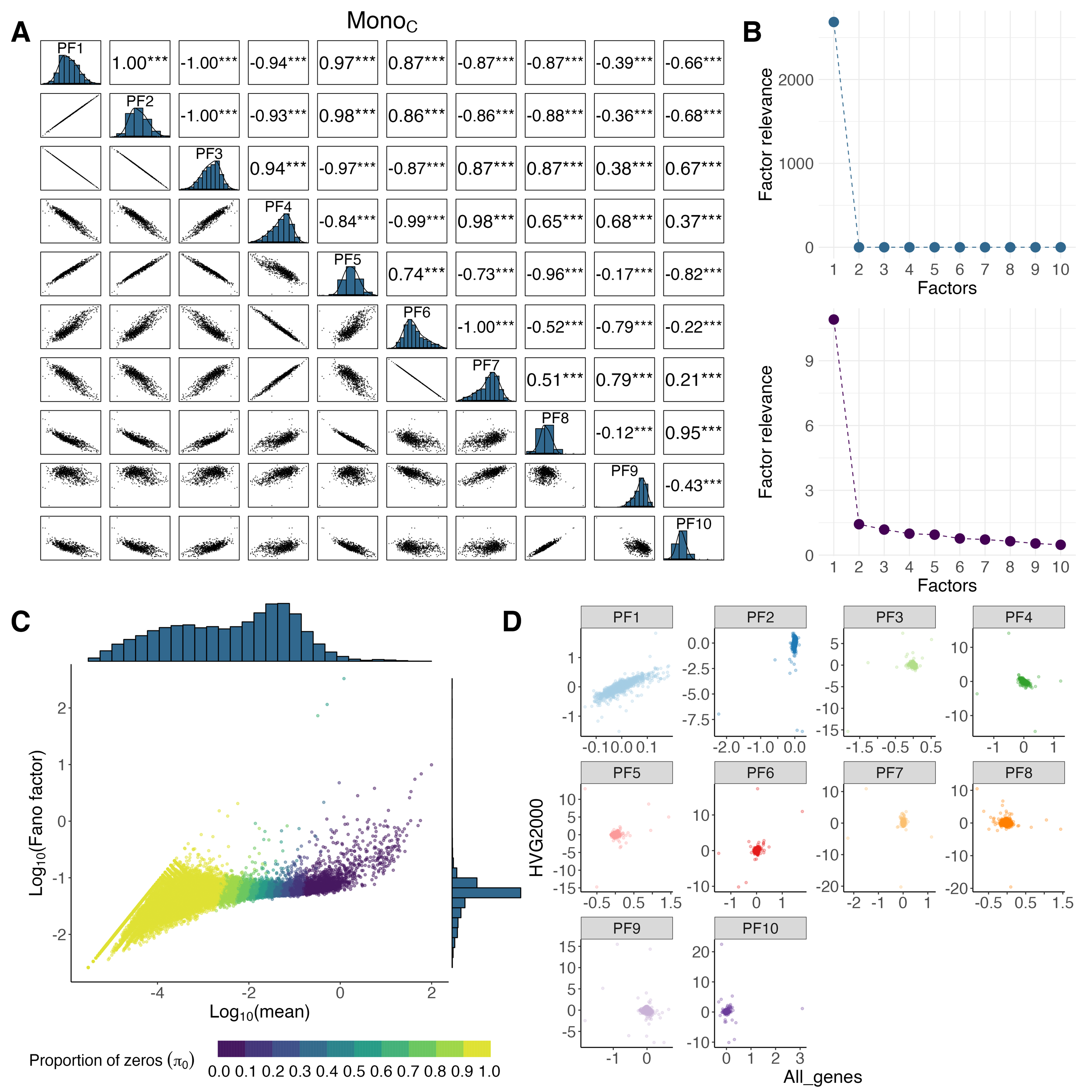

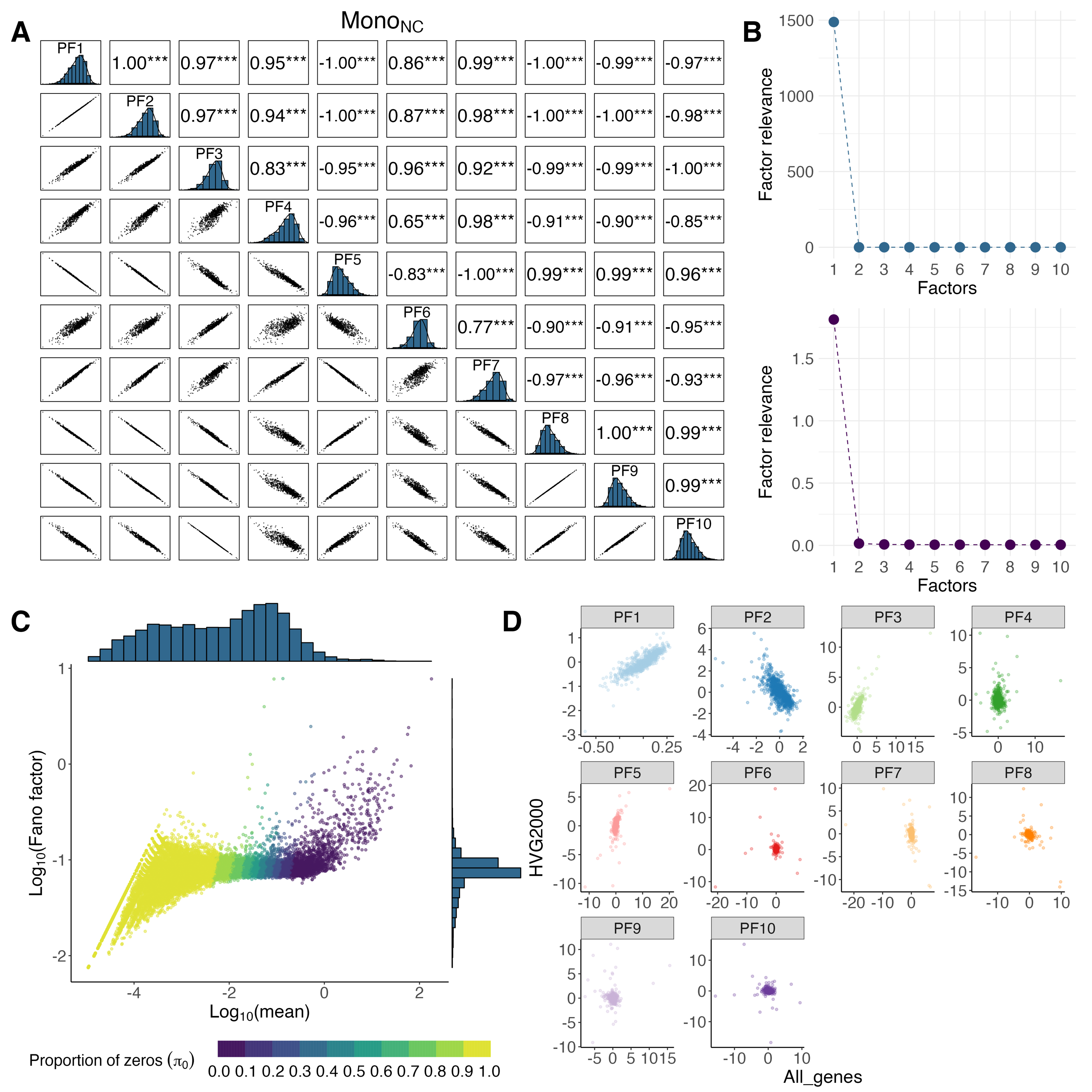

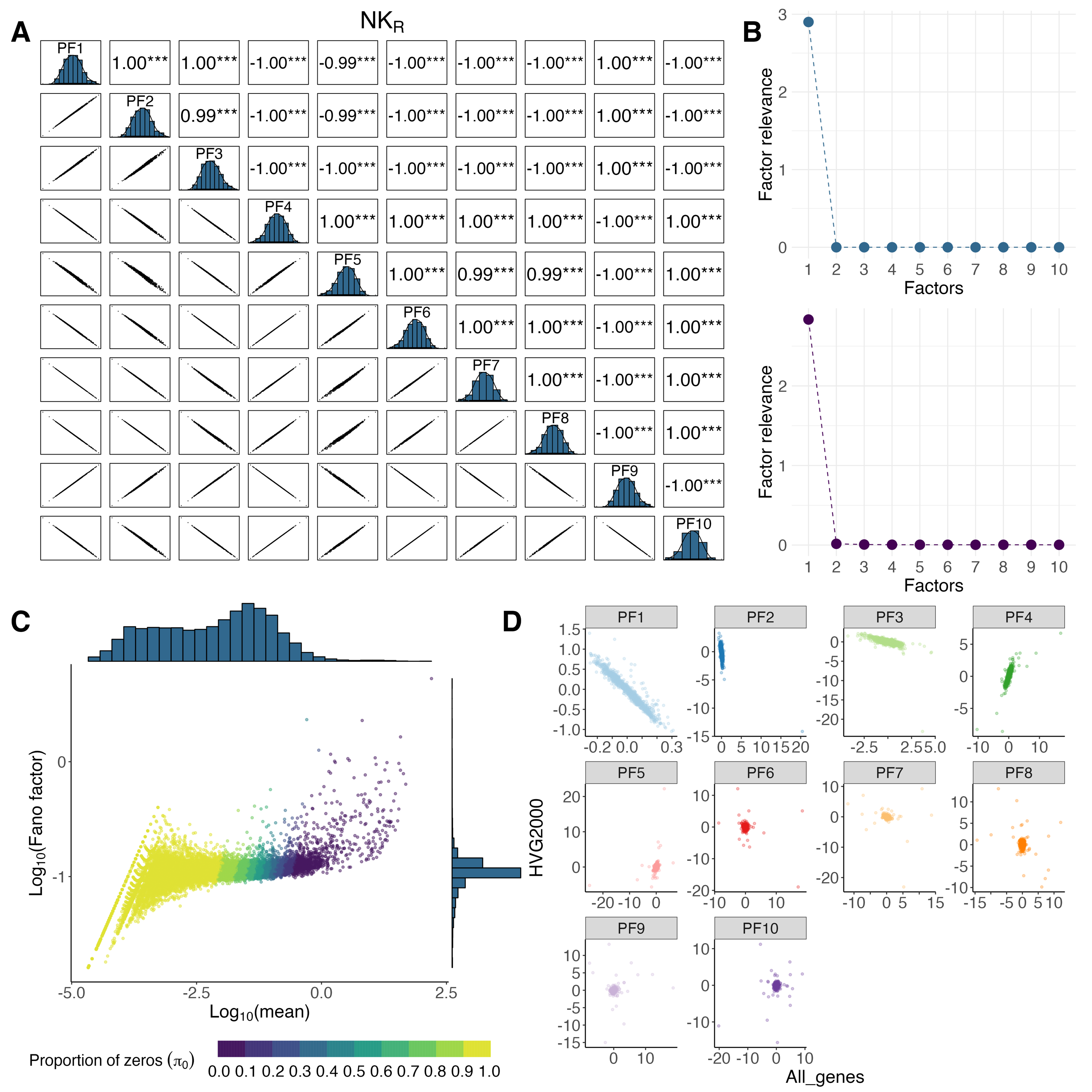

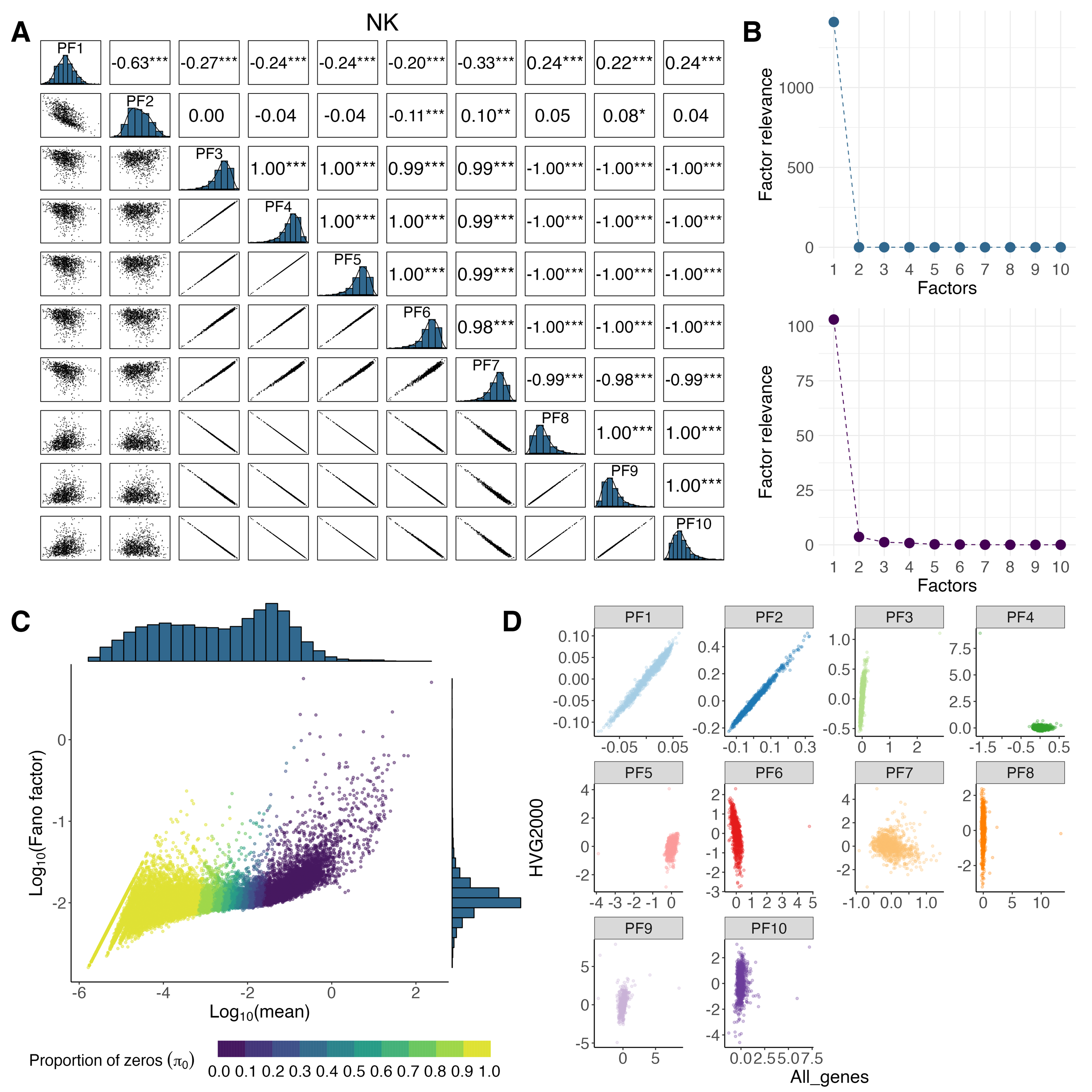

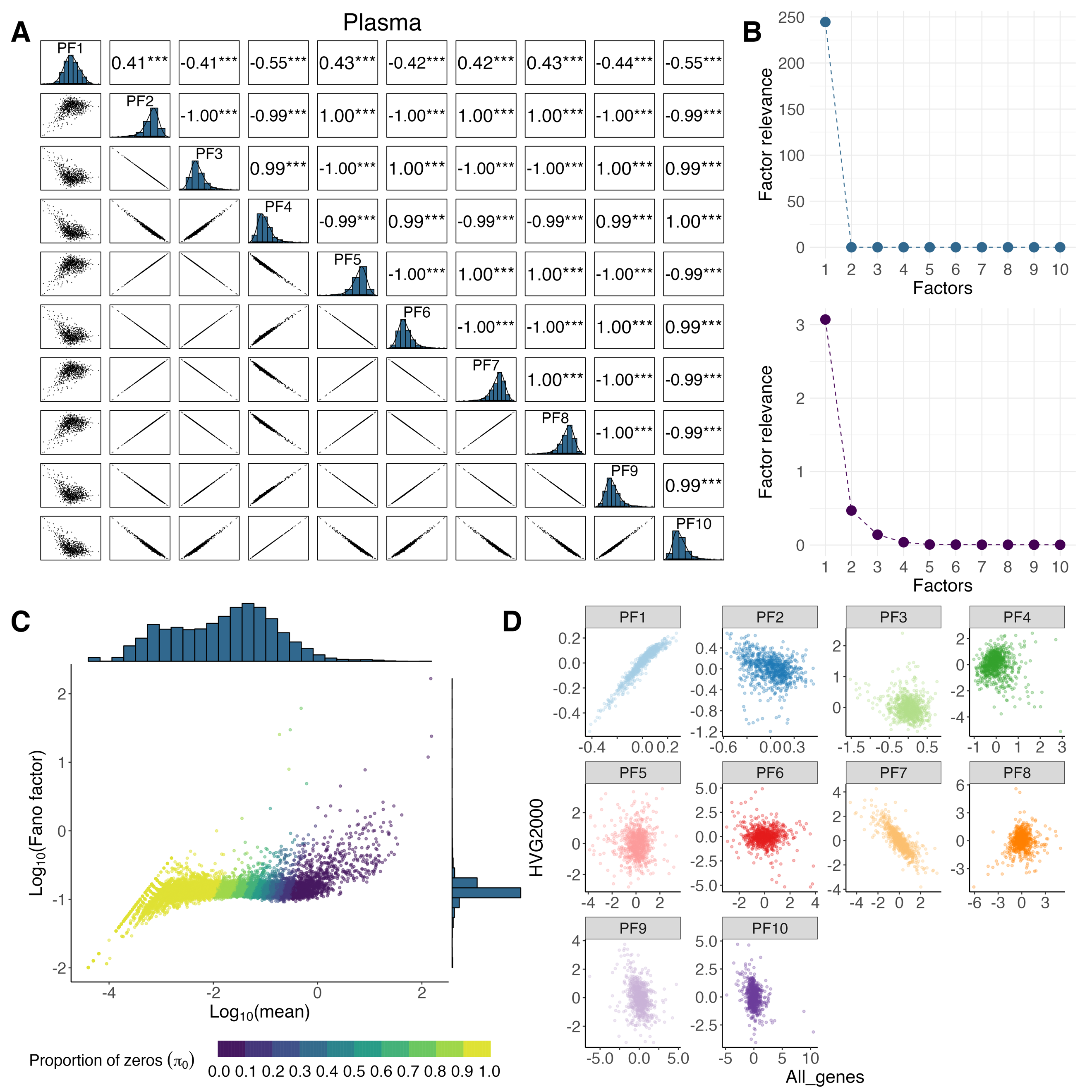
**

**Fig. S1. Correlation among inferred PEER factors and global intra-individual mean-variance dependence**. The figure legend is the same as **Figure 1**. **A,** Pair-wise correlation plot among the first 10 PFs generated from single-cell expression in CD4_NC_ cells without any QC (option #1). The upper triangle panel shows the pair-wise estimates of Pearson's correlation, and the bottom triangle panel shows the pair-wise scatter plot between the PFs. The diagonal panel shows the distribution of each PF. The significance of the correlation test is annotated by * *p*-value ≤ 0.05, ** ≤ 0.01, *** ≤ 0.001. **B**, Diagnostic plot of the factor weights without any further QC on the pseudo-bulk matrix (option #1, upper panel) and option #11 QC (lower panel). **C**, Relationship between intra-individual pseudo-bulk mean and Fano factor per gene. Both axes are Log10 transformed. The colour of the dots indicates the proportion of zero expression across individuals ($\pi_{0}$) for each gene. **D**, scatter plot of first 10 PEER factors generated from all genes against those from top 2000 HVGs (option #11 vs option #12). PF: PEER factor, QC: quality control, HVGs: highly variable genes.

**
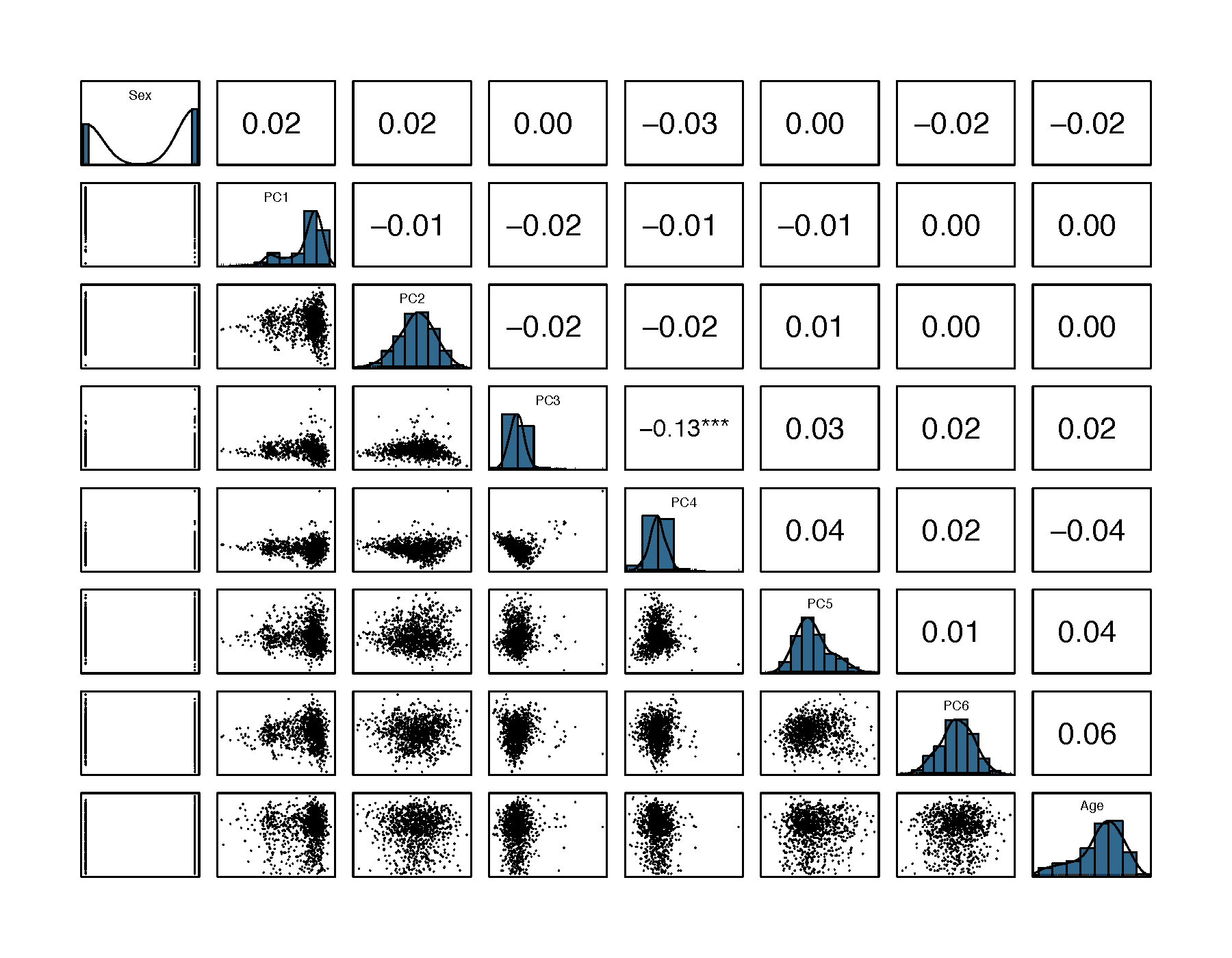
**

**Fig. S2. Correlation among known covariates, including sex, age, and first six genotype PCs**. The lower triangle denotes the scatter plot of pair-wise variables, and the red curve denotes the ﻿correlation ellipse. The upper triangle indicates the pair-wise estimate of Pearson's correlation coefficient *r*. The significance of the correlation test is annotated by * *p*-value ≤ 0.05, ** ≤ 0.01, *** ≤ 0.001. The diagonal panel represents the distribution of each know covariate. PC: principal components.

**Fig. S3. Different transformations of highly expressed and lowly expressed genes**. **A-B**, the x-axis, is the single-cell expression of highly expressed *FTH1* across individuals in CD4_NC cells. The y-axis is the log(x+1) transformed or RINT transformed expression. **C-D**, The same for lowly expressed *PMCH*. The grey dashed line represents the diagonal line of the coordinate panel.


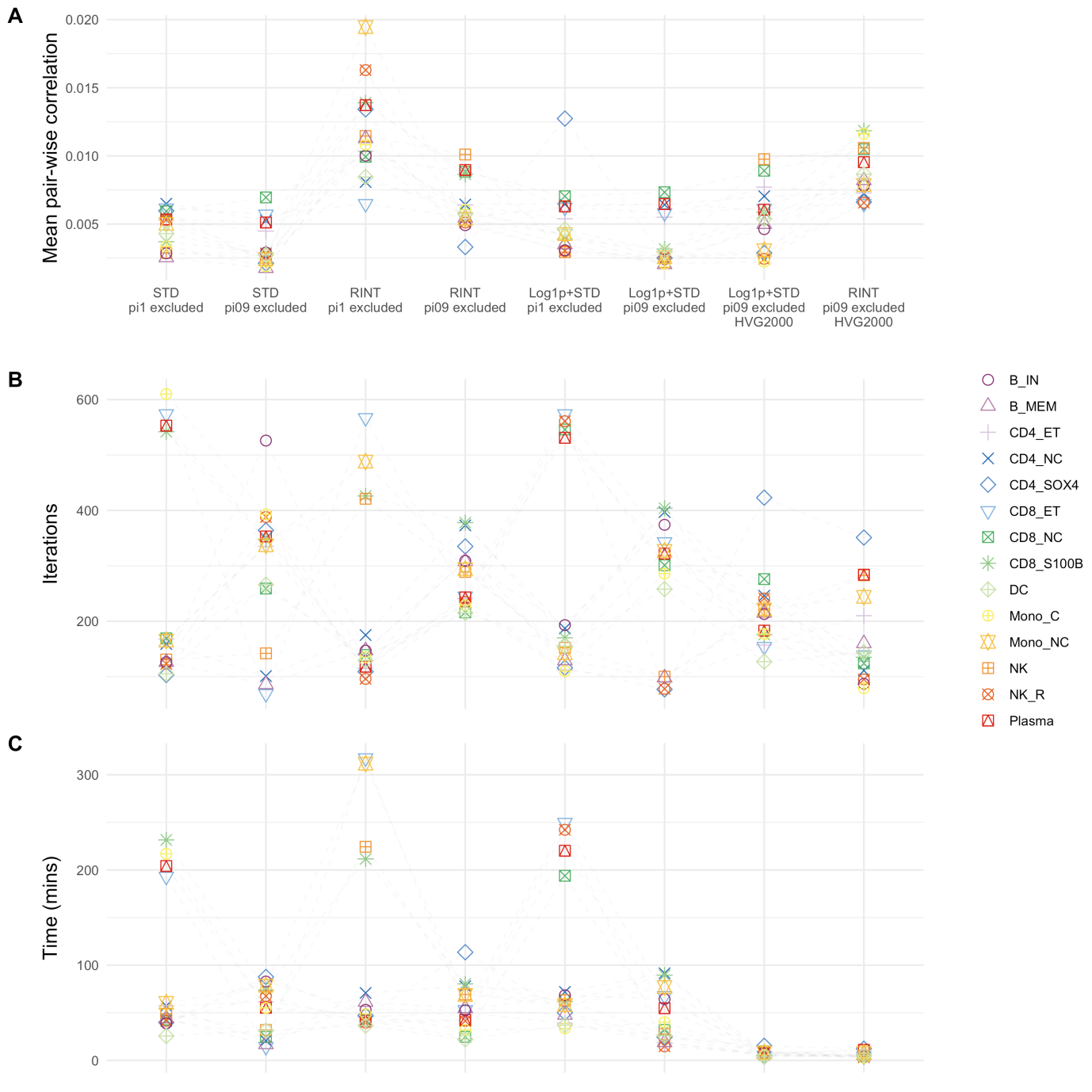


**Fig. S4. Performance of the 8 candidate QC options of input matrix for PEER factor generation**. **A**, The mean pair-wise correlation among the first 10 PFs. Each colour and shape represent a specific cell type. The x-axis corresponds to different QC options on the pseudo-bulk matrix. **B**, Number of iterations required for the algorithm to converge. **C**, Time to generate 50 PFs.


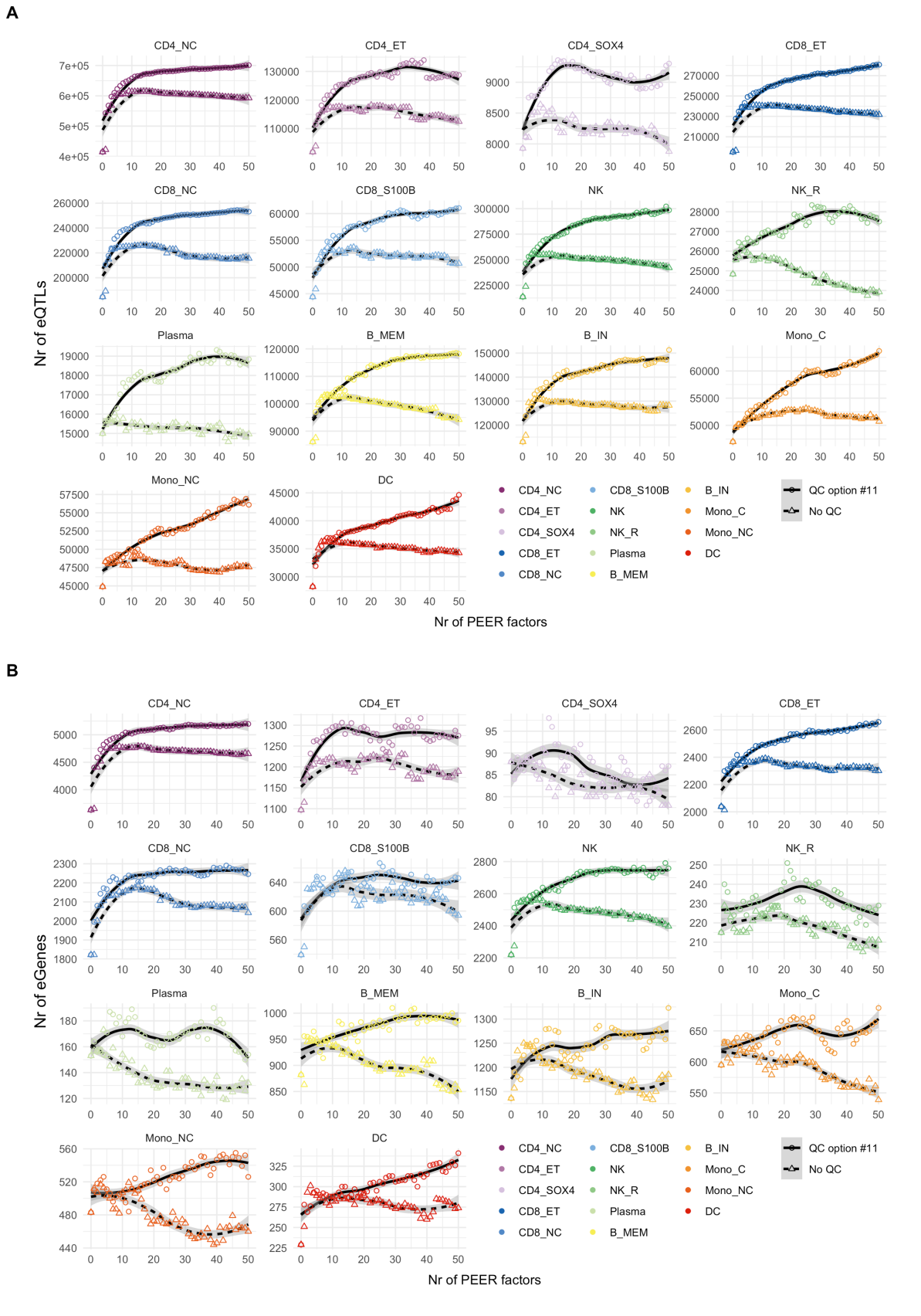


**Fig. S5. Sensitivity test for eQTLs and eGenes discovery power between no QC and QC option #11**. The y-axis represents the number of eQTLs (Panel **A**) or the number of eGenes (Panel **B**). Both x-axes denote the number of PEER factors fitted as covariates in the association model. Local regression is fitted for each QC option. The shape of each scatter point and the type of the fitted lines indicate whether using QC option #11 (circle, solid line) or no QC (triangle, dashed line) on the pseudo-bulk matrix to generate PEER factors. QC option #11 includes the steps to exclude genes with $\pi_{0}\geq0.9$, log(x+1) transformed and standardised the pseudo-bulk mean counts per gene.


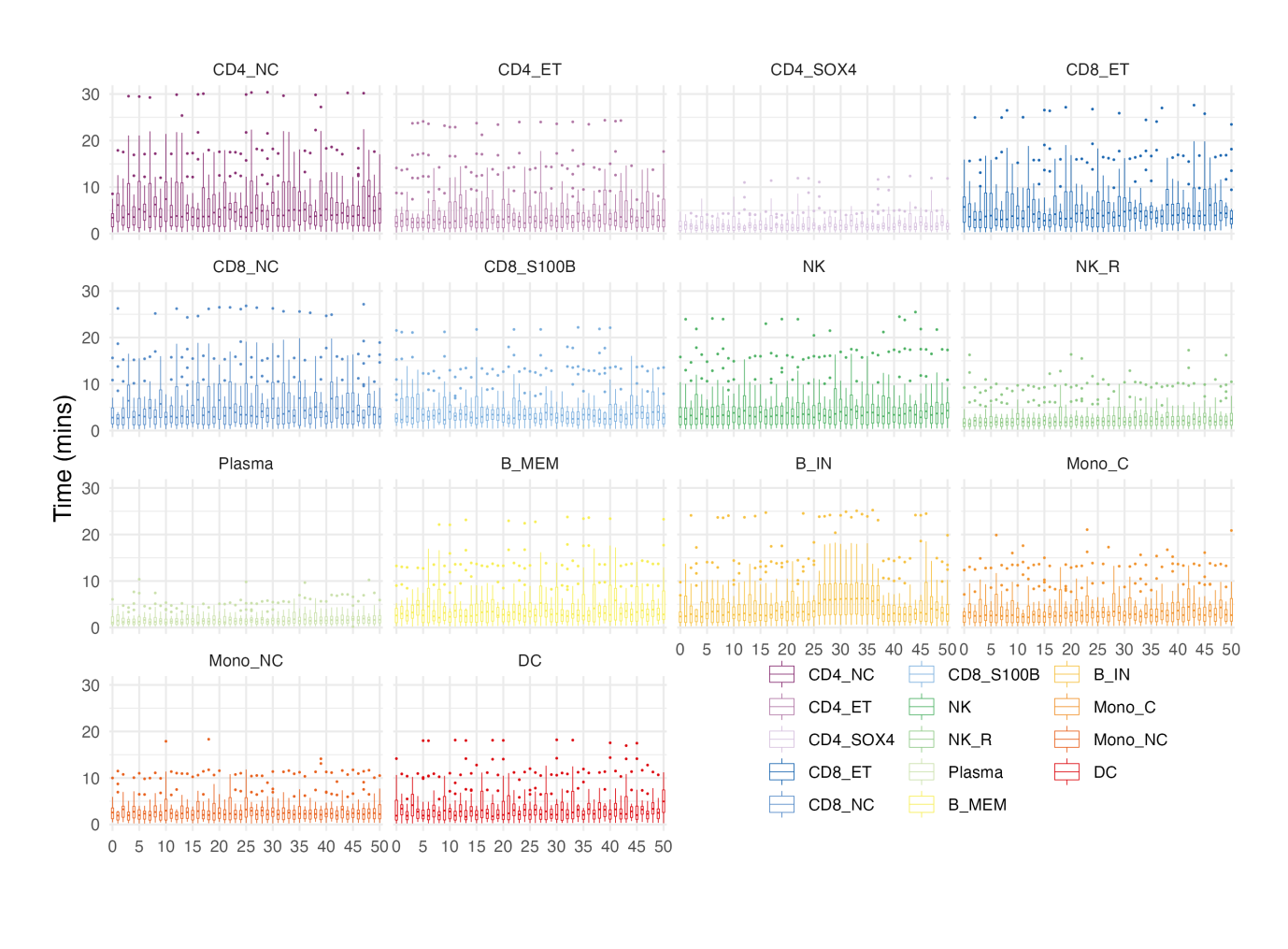
**Fig. S6. The computational time for eQTL association analysis when adjusting a different number of PEER factors**. The x-axis indicates the number of PEER factors fitted in the eQTL association model by Matrix eQTL software. The y-axis indicates the elapsed time of the association test for each chromosome. The boxplot represents the distribution of the elapsed time for 1-22 chromosomes separately.


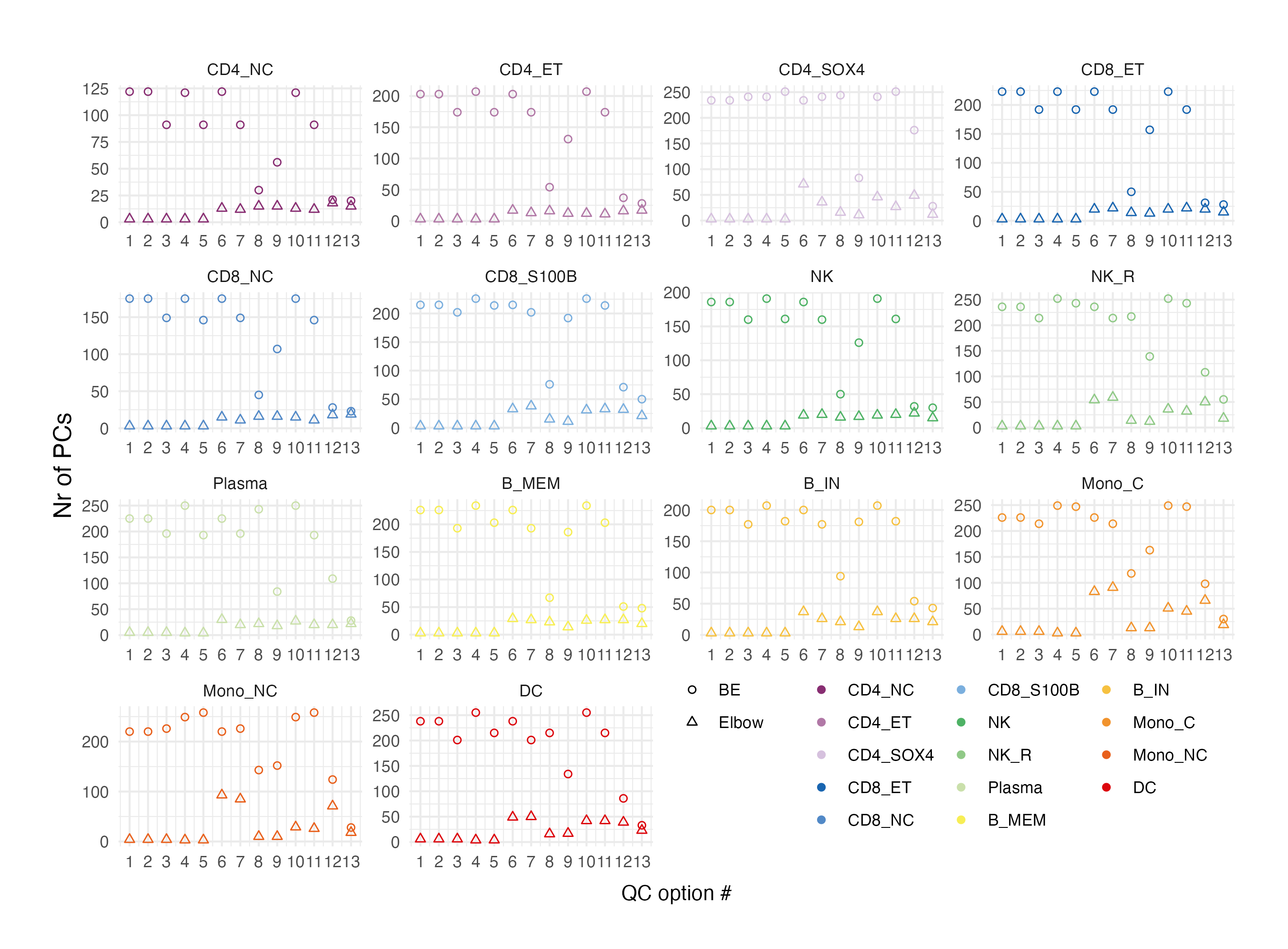


**Fig. S7. Detection of the optimal number of PCs**. This figure compares two methods to select the optimal number of PCs. BE (circle) indicates Buja and Eyuboglu algorithm, and Elbow (triangle) indicates the automatic elbow detection method. The x-axis indicates the QC options 1-14. The y-axis indicates the optimal number of PCs selected by the methods.


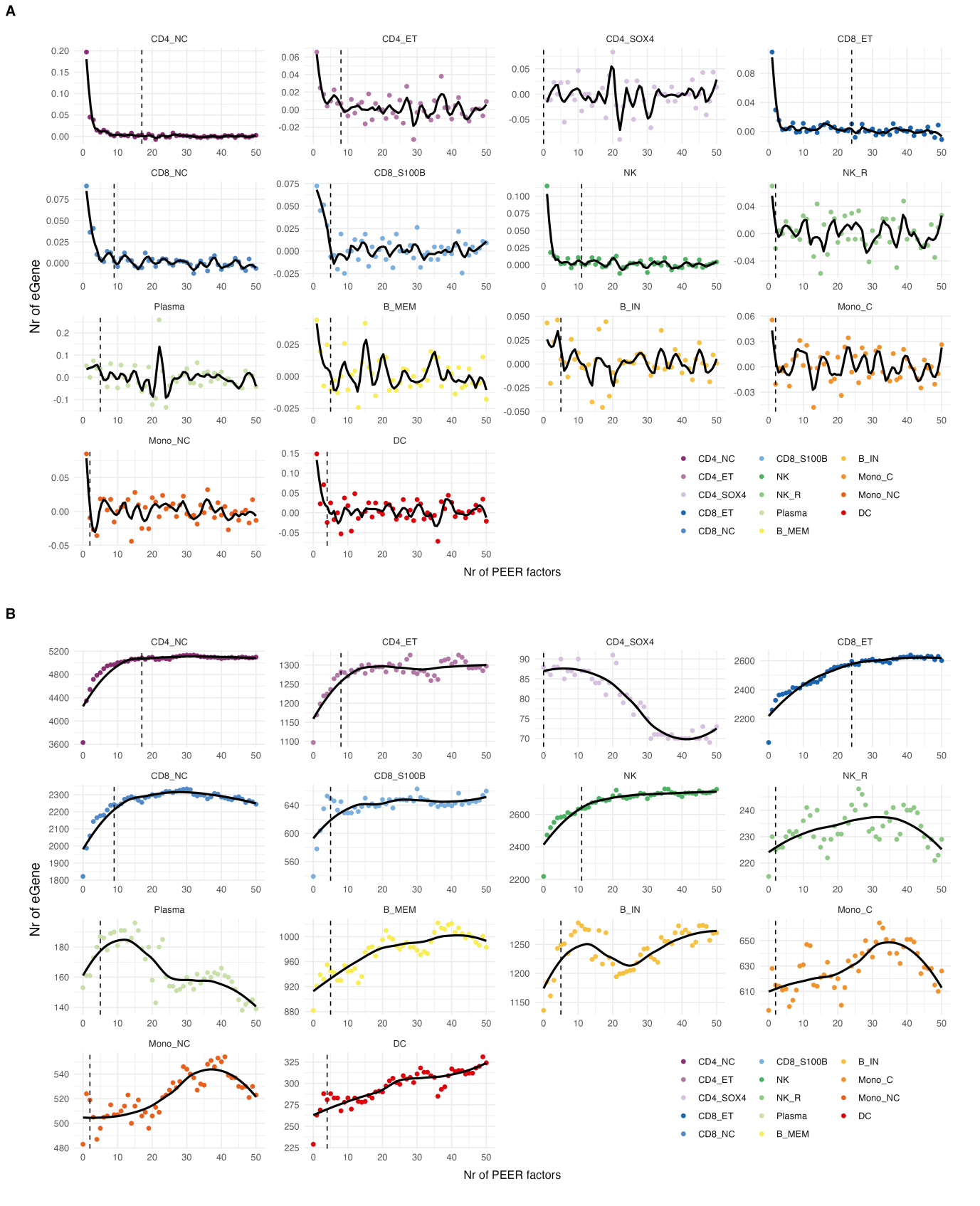


**Fig. S8. Detection of the optimal number of PEER factors using the local greedy algorithm**. This figure shows our local greedy algorithm (vertical dashed line) to choose the optimal number of PEER factors fitted in the eQTL association model. The PEER factors are generated with HVG2000 using QC option #12 as an example because it achieves similar power as using all genes but much quicker. The x-axis indicates the number of PEER factors. (**A**) The y-axis indicates the percentage change of the number of eGene detected when incrementally increasing the number of PFs fitted; The black trend curve line is fitted using loess function with *span* parameter set to 0.15. (**B**) The y-axis indicates the number of eGene detected.


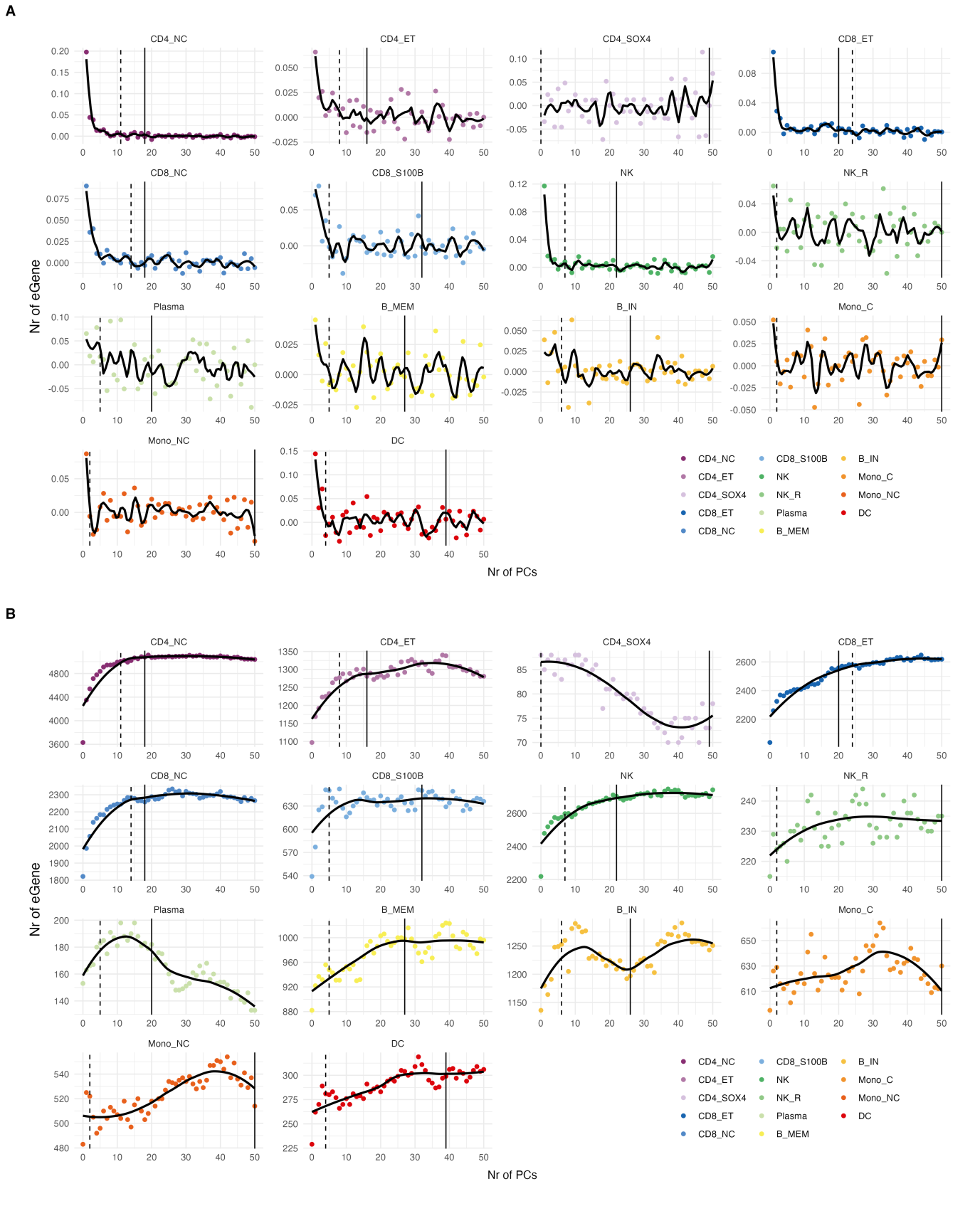


**Fig. S9. Detection of the optimal number of PCs using elbow detection method and the local greedy algorithm**. This figure compares the elbow detection method (vertical solid line) and local greedy algorithm (vertical dashed line) to choose the optimal number of PCs fitted in the eQTL association model. The optimal numbers of PCs returned by the elbow detection method for CD4_SOX4_, NK_R_, Mono_C_, Mono_NC_ are larger than 50 but marked as 50. The PCs are generated with HVG2000 using QC option #12 as an example. The x-axis indicates the number of PCs. (**A**) The y-axis indicates the percentage change of the number of eGene detected when incrementally increasing the number of PCs fitted; The black trend curve line is fitted using loess function with *span* parameter set to 0.15. (**B**) The y-axis indicates the number of eGene detected.

**Fig. S10. The correlation plot of fibroblast and iPSC clusters from Drew et al**. Here shows the correlation plot of four iPSC clusters. The four clusters correspond to the cell population classified by the relative activity of the following regulating transcription factors (cluster 1-4 = BRF2^+^, ATF2^+^, HIC2^+^ and CEBPG^+^). The lower triangle indicates the estimate of the correlation coefficient. The circles in the upper triangle show the size and sign of the correlation by circles. The blue circle indicates a positive correlation, and the red circle indicates a negative correlation. The correlation in fibroblast data was negligible (most correlation coefficients < 0.003), and thus was not shown here.


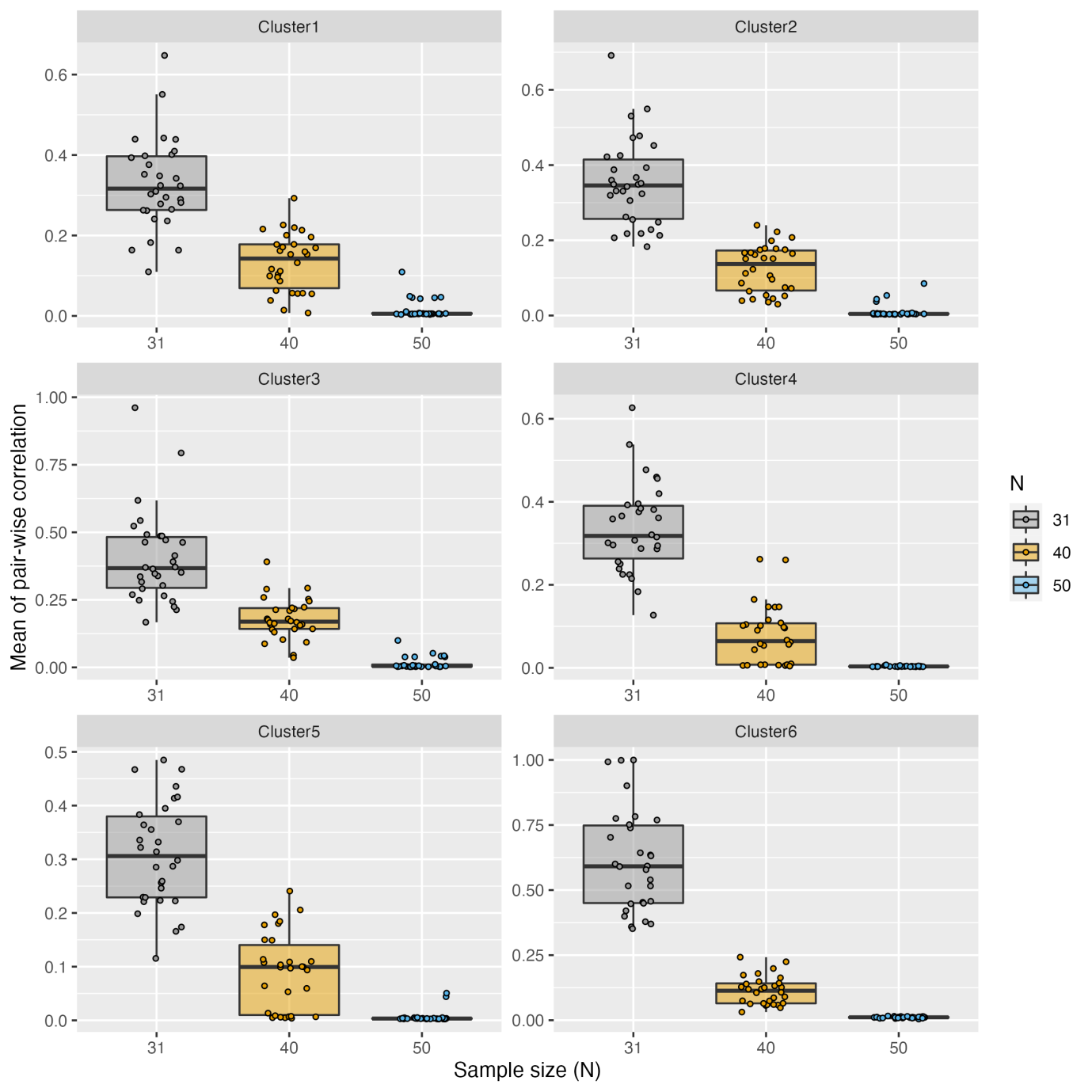


**Fig. S11. Distribution of mean pair-wise correlation coefficient among PEER factors in down-sampling of fibroblast clusters**. The x-axis indicates the sample size of three down-sampling tests annotated by a different colour. The y-axis indicates the mean of 45 pair-wise correlations (absolute value) among the first 10 PEER factors. Each dot represents one of the 30 replicates. Each panel shows one of the six clusters in the fibroblast data. The six clusters correspond to the cell population classified by the relative activity of the following regulating transcription factors (cluster 1-6 = SIX5^+^, HOXC6^+^, ATF1^+^, TEAD2^+^, KLF10^+^ and RXRB^+^).


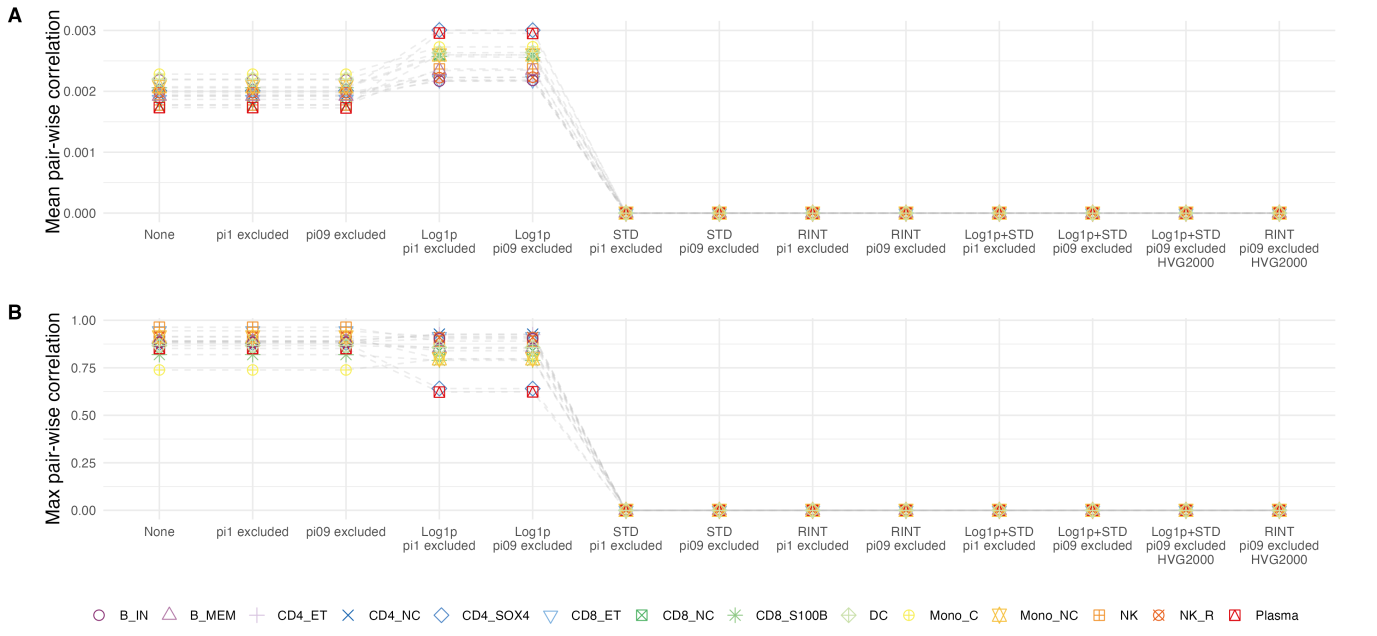


**Fig. S12. Performance of different QC options on the generation of expression PCs**. (**A**), The mean pair-wise correlation among the first 50 PCs. Each colour and shape represent a specific cell type. (**B**), The maximum pair-wise correlation among the first 50 PCs. The axis-s indicate 13 QC and transformation options. Full explanations of the options can be found in the **Methods** section. All the correlations in options 6-14 are zeros (the empirical correlation estimates are 1.6E-15 ~ 4.5E-14 in the computer). The R function of PCA, *prcomp* sets the “center” and “scale” as default, but in our analysis, we want to test our QC options and their performance under extreme cases, so we manually turned off these two flags when generating PCs.


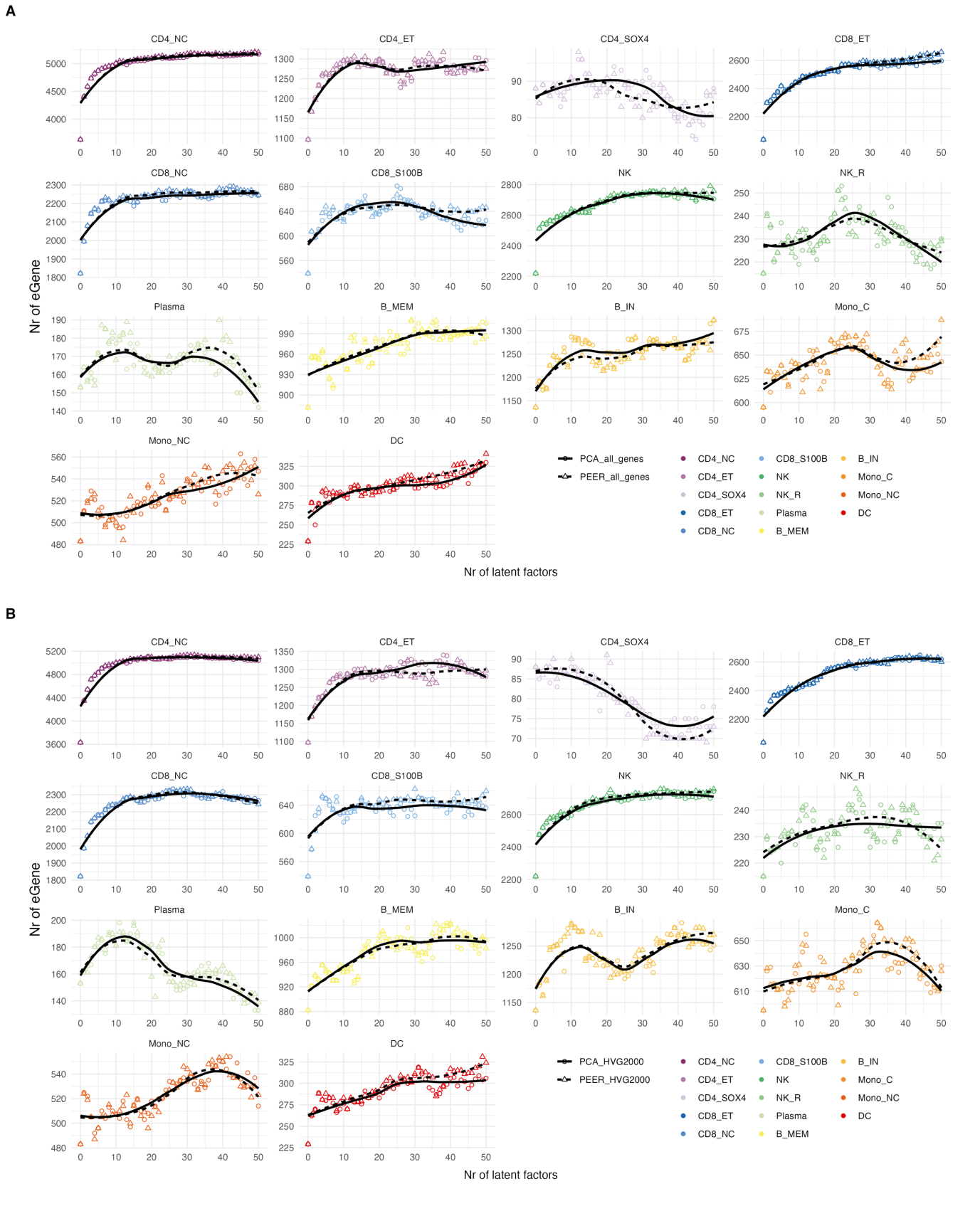


**Fig. S13. Sensitivity test for eGene detection using a different number of latent variables inferred from PCA and PEER**. The x-axis denotes the number of latent variables fitted as covariates in the association model. The y-axis represents the number of eGenes with at least one eQTL at local FDR < 0.05. The scatter shape is a circle for PCA and a triangle for PEER. The solid line is the LOESS curve for PCA, and the dashed line is the LOESS curve for PEER. Panel **A** indicates the comparison by using all genes to infer the latent variables, and **B** by using HVG2000.

| **Cell type** | ***N*** | **All genes**  (option #11) | | **HVG2000**  (option #12) | |
| --- | --- | --- | --- | --- | --- |
|  |  | **Prop.** | **Nr. of PF at peak** | **Prop.** | **Nr. of PF at peak** |
| B_IN | 980 | 0.165 | 50 | 0.133 | 10 |
| B_MEM | 980 | 0.145 | 29 | 0.159 | 36 |
| CD4_NC | 980 | 0.434 | 49 | 0.414 | 32 |
| CD4_ET | 980 | 0.201 | 38 | 0.209 | 28 |
| CD4_SOX4 | 857 | 0.114 | 12 | 0.034 | 20 |
| CD8_NC | 980 | 0.257 | 43 | 0.281 | 30 |
| CD8_ET | 980 | 0.304 | 50 | 0.295 | 41 |
| CD8_S100B | 979 | 0.238 | 25 | 0.230 | 30 |
| DC | 967 | 0.489 | 50 | 0.445 | 49 |
| Mono_C | 967 | 0.155 | 50 | 0.116 | 32 |
| Mono_NC | 932 | 0.149 | 46 | 0.147 | 41 |
| NK_R | 968 | 0.167 | 21 | 0.154 | 26 |
| NK | 980 | 0.258 | 49 | 0.243 | 35 |
| Plasma | 795 | 0.242 | 39 | 0.281 | 15 |

**Table S1. The maximum of eGene detection power gain by incorporating PEER factors**. The column "Prop." indicates the power gain, and "Nr. of PF at peak" is the number of PFs fitted at the maximum number of eGenes. The power gain is measured by the proportion of new eGenes that can be maximised by fitting PEER factors compared to the number when no PEER factors are fitted. *N* indicates the sample size for each cell type.
