# Supplementary material for: Pitfalls and opportunities for applying latent variables in single-cell eQTL analyses
Source: Genome Biol. 2023 Feb 23;24:33. doi: 10.1186/s13059-023-02873-5 (PMC9948363; doi:10.1186/s13059-023-02873-5)
Supplement: Supplementary file 2 — Additional file 2. Review history. [file 13059_2023_2873_MOESM2_ESM.docx]

Review History

**First round of review**

**Reviewer 1**

**Were you able to assess all statistics in the manuscript, including the appropriateness of statistical tests used?**

Yes, and I have assessed the statistics in my report.

**Comments to author:**

This paper examines the data processing steps prior to PEER factor calculation and its influence on single-cell eQTL data analysis. A series of analyses showed that selecting SVGs and performing data transformation steps are important for obtaining valid PEER factors and for achieving powerful eQTL association results. Diagnostic guidelines are provided for improving the robustness in generating PEER factors for single-cell eQTL association analyses. Overall, the paper is well written, and the topic is timely. I only have a couple major comments that hopefully would help improve the quality of the paper further:

1. Many bulk RNAseq eQTL mapping studies directly use the principal components (PCs), instead of PEER factors, to control for confounding factors (e.g. PMC3089435; PMC4383332). Some bulk eQTL mapping studies also use ComBat to control for confounding factors (e.g. PMC7118581). Unlike PEER factors, a key benefit of PCs is that PCs are all orthogonal to each other with zero correlation between them. I was wondering if the same data processing steps that worked well for PEER factor analysis would also work well when using PCs (or ComBat factors) for single-cell eQTL mapping, or whether we need a different set of data processing recommendations when using PCs. In addition, should we use PCs or PEER factors for single-cell eQTL mapping, if we want to maximize the number of eGene discovery?

2. One recommendation of the present study is to perform HVG selection before PEER factor analysis. A key computational benefit of using HVGs is that it leads to a 6.2x speed gain for PEER factor analysis. However, I think it is important to mention in the paper that the computational cost of PEER factor analysis often only represents a small fraction of the overall computational cost for eQTL analysis. Afterall, the eQTL association analysis itself on testing millions of pairs of SNPs-genes usually dominates the computational cost of eQTL analysis.

In addition, as stated in the Introduction, previous studies have to either perform repetitive eQTL mapping on a range of PEER factor numbers or determine the number of PEER factors based on sample size in an ad hoc fashion. Performing repetitive eQTL mapping on a range of PEER factor numbers is computationally challenging for large data, while choosing the number of PEER factors based on sample size alone may not be effective. There is a recent computational method, called the effect size Correlation for COnfounding determination (ECCO; PMCID: PMC8058772), solves the computational issue associated with determining the optimal number of PEER factors. In particular, ECCO uses a new strategy of examining the effect size estimate correlation to determine the number of optimal PEER factors used for eQTL mapping. Consequently, one no longer needs to perform repetitive eQTL mapping or rely on sample size to determine the optimal number of PEER factors, thus leading to substantial improvement of computational efficiency. I was wondering whether this approach of ECCO can be directly applied to single cell eQTL mapping analysis, to solve some of the computational hurdles mentioned in the paper. This strategy may also allow the authors to explore larger number of PEER factors beyond 50 listed in Figure 2C, where the number of eGenes does not appear to reach a saturation point at 50 for quite a few cell types (e.g. CD9_ET NK, Mono_NC, and DC).

**Reviewer 2**

**Were you able to assess all statistics in the manuscript, including the appropriateness of statistical tests used?**

Yes, and I have assessed the statistics in my report.

**Comments to author:**

This paper studies the use of PEER factors in single-cell pseudo-bulk eQTL analysis and has the following three main conclusions: first, without proper quality control and/or data transformation, PEER factors can be highly correlated among themselves; second, the number of PEER factors that maximizes discoveries depends on the specific data set at hand; third, using highly variable genes to compute PEER factors produces similar results but saves computational time compared to using all genes to compute PEER factors. These conclusions, especially the first one, are consistent with the findings in Zhou et al. [1], where it is reported that PEER factors can be highly correlated and thus fail to capture important variance components in certain bulk 3'aQTL data.

Main comments:

1. Previous studies have reported that PCA performs better than PEER in both bulk [1] and pseudo-bulk eQTL analysis [2]. It would be informative to include PCA in the analyses in this paper. In particular, PCs are always uncorrelated; PCA is much faster than PEER (even if PEER is run on only the highly variable genes); and PCA offers ways of choosing the number of inferred covariates that are not based on the resulting discoveries [1]. Since the authors are proponents of choosing the number of PEER factors by maximizing the number of discoveries (though this practice is theoretically questionable [1] because it may lead to false discoveries), I suspect that the optimal number of PCs chosen in the same way may lead to as many or more discoveries.

2. The second and third issues pointed out on Page 2 (non-normal distribution and mean-variance dependency) also apply to bulk RNA-seq data. They should not be treated as specific to single-cell RNA-seq data.

3. There are different ways to run PEER, e.g., with known covariates and without known covariates; see [1] for detail. The authors need to consider this issue. Currently the authors generate PEER factors "while including sex, age, and six genotype PCs as covariates".

Minor comments:

1. Line 2-7, "PEER is a method that implements a Bayesian framework to estimate the latent variables and jointly learn the contribution to the gene expression variability from genotype, known factors, and hidden factors." This sentence may be edited to reflect that although the original PEER paper claims this, currently the PEER package cannot take genotype data as an input (and known factors are optional inputs) [3].

2. Line 2-17, "the data structure will be assimilated into the bulk RNA-sequence data": do the authors mean that the data structure is similar to bulk data?

3. Lines 2-17 to 2-24: the writing of this paragraph may be improved; in particular, the noun phrases may be unified to help more readers understand. From my understanding, pseudo-bulk expression data are obtained from scRNA-seq data, and bulk expression data are obtained from bulk RNA-seq data. And the main purpose of this paragraph is to introduce the differences between pseudo-bulk expression data and bulk expression data. Ideas for improvement include: in Line 19, "single-cell expression matrix" may be better replaced by "pseudo-bulk expression matrix"; in Line 20, "single-cell and bulk RNA data" may be better replaced by "pseudo-bulk expression data and bulk expression data"; in Line 23, "the underlying distribution of gene expression across cells follows either negative binomial (NB) or zero-inflated NB (ZINB) distributions" - the authors may want to clarify that this statement is regarding the pre-aggregation single cell data.

4. Lines 4-3 to 4-10, "This is consistent with the observation that…": the logic of this reasoning seems unclear to me.

5. Line 4-1: for the benefit of the reader, point out where these 13 options are listed in full. The Methods section doesn't seem to contain this information.

6. Line 4-24: what does the "skewness of genes" mean?

References:

[1] Heather J. Zhou, Lei Li, Yumei Li, Wei Li, and Jingyi Jessica Li. PCA outperforms popular hidden variable inference methods for molecular QTL mapping. bioRxiv, 2022. https://doi.org/10.1101/2022.03.09.483661.

[2] Anna S. E. Cuomo, Giordano Alvari, Christina B. Azodi, single-cell eQTLGen consortium, Davis J. McCarthy, and Marc Jan Bonder. Optimizing expression quantitative trait locus mapping workflows for single-cell studies. Genome Biology, 22(1):188, 2021.

[3] Heather J. Zhou. Capturing hidden covariates with linear factor models and other statistical methods in differential gene expression and expression quantitative trait locus studies. UCLA Electronic Theses and Dissertations, 2022. https://escholarship.org/uc/item/2rq72420.

**Reviewer 3**

**Were you able to assess all statistics in the manuscript, including the appropriateness of statistical tests used?**

Yes, and I have assessed the statistics in my report.

**Comments to author:**

Xue et. al. empirically evaluated different QC steps on pseudo-bulk matrix from single cell gene expression data for obtaining PEER factors, to deal with the issue of highly correlated PEER factors due to the violation to the assumptions of PEER methods. Sensitivity analysis was ran to compare between QC and no QC. Performance on number of eGenes under different number of PEER factors is also shown for different cell types when using all genes or just the top 2000 most variable ones.

I think the methods are appropriate to the aims of the study and are well described. I just have some minor concerns:

From the methods section, we can tell that when using the top 2000 HVGs, these genes are chosen before transformation and scaling. It might be better to also make this clear somewhere in the main body of the paper. Otherwise, the ordering of the five options in line 12~18 on page 6 and the x axis labels for the last two combinations in Figure 2(A) might mislead the readers to think these top 2000 genes are chosen after the standardization.

Are the data files available for potential readers to directly replicating the results using the provided code?

This one is likely a typo. Line 10 on page 7, "and the gains of made in number of detected eGenes were also similar", this sentence seems not smooth. Should "of made" be deleted?

**Authors Response**

**Point-by-point responses to the reviewers’ comments:**

Reviewer #1:

This paper examines the data processing steps prior to PEER factor calculation and its influence on single-cell eQTL data analysis. A series of analyses showed that selecting SVGs and performing data transformation steps are important for obtaining valid PEER factors and for achieving powerful eQTL association results. Diagnostic guidelines are provided for improving the robustness in generating PEER factors for single-cell eQTL association analyses. Overall, the paper is well written, and the topic is timely. I only have a couple major comments that hopefully would help improve the quality of the paper further:

1. Many bulk RNAseq eQTL mapping studies directly use the principal components (PCs), instead of PEER factors, to control for confounding factors (e.g. PMC3089435; PMC4383332). Some bulk eQTL mapping studies also use ComBat to control for confounding factors (e.g. PMC7118581). Unlike PEER factors, a key benefit of PCs is that PCs are all orthogonal to each other with zero correlation between them. I was wondering if the same data processing steps that worked well for PEER factor analysis would also work well when using PCs (or ComBat factors) for single-cell eQTL mapping, or whether we need a different set of data processing recommendations when using PCs. In addition, should we use PCs or PEER factors for single-cell eQTL mapping, if we want to maximize the number of eGene discovery?

*Reply: Thank you for highlighting the comparison between PCs/ComBat and PEER factors, which we agree are commonly used to account for ‘hidden’ variation in eQTL association testing. We have now included an analysis of PCs in addition to PEER factors.*

*We have used “PCAForQTL” (Zhou et al. bioRxiv, 2022) to generate the expression PCs for the OneK1K cohort in each of 14 cell types, following the same analysis strategy we used for PEER factors. The PCs inferred without proper QC and scaling also showed spurious correlations, but to a lesser extent than PEER factors (Supp Figure 10). We then performed the eQTL sensitivity analyses with incremental adjustment for PCs 0-50 and compared the number of eGenes detected by PEER factors. The number of eGenes is generally consistent, whether using PCs or PEER factors, and the trend curve of the incremental number of latent variables overlap with each other (Supp Figure 11). We have included all these results and edited the manuscript throughout.*

*A recent review paper (Cuomo et al. 2021) published in Genome Biology (also mentioned by reviewer #2) found the PCA performs better than PEER but only between 5-15th factors adjusted. Rather, when including more than 20 factors, PEER outperforms PCA. This comparison was only applied to iPSC data with 125 donors (Cuomo et al. 2020. Nat Comms) and only tested up to 25 factors. We conclude that there is no major difference in PCs and PEER factors in maximising the number of eGene discoveries, but both methods need more caution during the QC and scaling when applied to single-cell data.*

2. One recommendation of the present study is to perform HVG selection before PEER factor analysis. A key computational benefit of using HVGs is that it leads to a 6.2x speed gain for PEER factor analysis. However, I think it is important to mention in the paper that the computational cost of PEER factor analysis often only represents a small fraction of the overall computational cost for eQTL analysis. Afterall, the eQTL association analysis itself on testing millions of pairs of SNPs-genes usually dominates the computational cost of eQTL analysis.

*Reply: We agree that it’s important to consider the computational resources of PEER calculation and correction as part of a larger eQTL analysis strategy. We have evaluated the computational cost for the main eQTL analysis for all datasets included in this paper. Using Matrix eQTL software, it typically took approximately 30 mins for the cis-eQTL association analysis to complete if we submit a batch job for 22 chromosomes for each cell type. Therefore, we have found that calculating PEER factors can be a significant fraction of the overall computational cost and possibly a bottleneck without proper optimisation. However, we recognise that this ratio is likely to be much more skewed toward the eQTL association analysis in bulk data. As a hypothetical, imagine scRNA-seq data on 10,000 individuals; it may take > 24 hours to calculate 50 PEER factors, while our QC + HVG strategy only would take 1~2 hours. One of the challenges in the analysis for PEER factors calculation is the inability to separate jobs into batches (i.e., chromosomes). We have added Supp Figure 6 describing the computational time for eQTL analyses given under different numbers of PEER factors, and edited the text to reflect this also.*

*On a different note, we acknowledge that the eQTL sensitivity test could be computationally intensive as it requires 22 * n PFs jobs for each cell type, and each job typically requires 5-15 GB of CPU memory). A simple way to reduce the computationally burden is to run the sensitivity test just for one chromosome (e.g., 1 or 22).*

In addition, as stated in the Introduction, previous studies have to either perform repetitive eQTL mapping on a range of PEER factor numbers or determine the number of PEER factors based on sample size in an ad hoc fashion. Performing repetitive eQTL mapping on a range of PEER factor numbers is computationally challenging for large data, while choosing the number of PEER factors based on sample size alone may not be effective. There is a recent computational method, called the effect size Correlation for COnfounding determination (ECCO; PMCID: PMC8058772), solves the computational issue associated with determining the optimal number of PEER factors. In particular, ECCO uses a new strategy of examining the effect size estimate correlation to determine the number of optimal PEER factors used for eQTL mapping. Consequently, one no longer needs to perform repetitive eQTL mapping or rely on sample size to determine the optimal number of PEER factors, thus leading to substantial improvement of computational efficiency. I was wondering whether this approach of ECCO can be directly applied to single cell eQTL mapping analysis, to solve some of the computational hurdles mentioned in the paper. This strategy may also allow the authors to explore larger number of PEER factors beyond 50 listed in Figure 2C, where the number of eGenes does not appear to reach a saturation point at 50 for quite a few cell types (e.g. CD8_ET, NK, Mono_NC, and DC).

*Reply: We thank the reviewer for suggesting the ECCO method and agree that strategies to determine the optimal number of PEER factors (or PCs) are very important. We have included commentary on using ECCO, but note that this method requires an additional quantitative phenotype with genetic determinants and was associated with gene expression levels, such as height, BMI, fasting glucose, and blood pressure. It should be noted also that this method assumes the residual errors to be independently and identically distributed from a normal distribution. This assumption will largely be satisfied in the bulk data, but we anticipate this is unlikely in single-cell RNA data because of the sparsity and even RINT transformation cannot make them normally or quasi-normally distributed (Supp Figure 3C-D).*

*However, recognising this important point, we implemented a commonly used method to decide the optimal number of PCs (Zhou et al. bioRxiv, 2022), the automatic elbow detection method (Methods and Supp Figure 7). We showed that for most cell types it returned a reasonable number of PFs but for CD4SOX4 this method identified a number of PFs that led to a lower number of eGene detected than that without fitting any PFs (Supp Figure 7B-C). This is the limitation of the elbow detection method as it unintentionally assumes a monotone increase of eGene along with the incremental fitting of the inferred latent variables. To overcome this, we proposed a new and simple strategy (named as local greedy algorithm) to calculate the optimal number of PEER factors required per cell type and help solve the observation that the saturation point varies in different cell types. First, we calculated the percentage change of eGenes with every additional PF / PC added. Then we performed a LOESS (locally estimated scatterplot smoothing) of the percentage change against the number of PFs / PCs (Supp Figure 7B). Instead of choosing the number of PFs/PCs, maximising the number of eGenes, we selected the number before the LOESS curve became negative. By this balanced strategy, most cell types will be only adjusted with 2~10 PFs or PCs rather than 20~50 but retain ~71% power gain of eGene discovery (Supp Figure 7C). The rationale behind this algorithm is that if the number of eGenes reaches saturation, the percentage change should be symmetrically spread around 0, so adding more factors is equivalent to adding more noises. Empirically, most LOESS curves after 10~20 factors were randomly distributed around 0.*

Reviewer #2:

This paper studies the use of PEER factors in single-cell pseudo-bulk eQTL analysis and has the following three main conclusions: first, without proper quality control and/or data transformation, PEER factors can be highly correlated among themselves; second, the number of PEER factors that maximizes discoveries depends on the specific data set at hand; third, using highly variable genes to compute PEER factors produces similar results but saves computational time compared to using all genes to compute PEER factors. These conclusions, especially the first one, are consistent with the findings in Zhou et al. [1], where it is reported that PEER factors can be highly correlated and thus fail to capture important variance components in certain bulk 3'aQTL data.

*Reply: We thank the reviewer for summarising our study and referencing relevant papers. We have addressed all the comments and discussed similarities and dissimilarities with the previous studies.*

Main comments:

1. Previous studies have reported that PCA performs better than PEER in both bulk [1] and pseudo-bulk eQTL analysis [2]. It would be informative to include PCA in the analyses in this paper. In particular, PCs are always uncorrelated; PCA is much faster than PEER (even if PEER is run on only the highly variable genes); and PCA offers ways of choosing the number of inferred covariates that are not based on the resulting discoveries [1]. Since the authors are proponents of choosing the number of PEER factors by maximizing the number of discoveries (though this practice is theoretically questionable [1] because it may lead to false discoveries), I suspect that the optimal number of PCs chosen in the same way may lead to as many or more discoveries.

*Reply: We thank the reviewer for making these suggestions, which concur with reviewer #1. In the revised manuscript, we have now included the PCA analysis compared to PEER factors and used the same QC pipelines to generate PCs. The PCs inferred without proper QC and scaling also showed spurious correlations but to a lesser extent than PEER factors (Supp Figure 10). We then performed the eQTL sensitivity analysis with incremental adjustment for PCs 0-50 and compared the number of eGenes detected with PEER factors. The number of eGenes detected is consistent whether using PCs or PEER factors, and the trend curves of the incremental number of latent variables overlap with each other (Supp Figure 11).*

*For choosing the number of inferred covariates, the methods used for PCA are not all PC-specific (Zhou et al. bioRxiv, 2022). This study compared two algorithms, automatic elbow detection method and Buja and Eyuboglu (BE) algorithm. After carefully reading the paper, we found the former can be directly applied to our PEER factors results since it only requires the information of the proportion of variance explained by each inferred covariate (similar to the factor relevance for each PF). Therefore, we have implemented this automatic elbow detection method to identify the optimal number of PEER factors or PCs for single-cell eQTL analysis (Supp Figure 7A). We showed that for most cell types it returned a reasonable number of PFs but for CD4SOX4 this method identified a number of PFs that led to a lower number of eGene detected than that without fitting any PFs (Supp Figure 7B-C). This is the limitation of the elbow detection method as it unintentionally assumes a monotone increase of eGene along with the incremental fitting of the inferred latent variables.*

*In terms of maximising the number of discoveries, we would like to clarify that we do not suggest choosing as many PEER factors as possible (like a globally greedy algorithm), and we are aware of the potential false discoveries. Instead, our suggestion is to balance the discovery and avoid potential overfitting by implementing an algorithm to identify the optimal number of PFs or PCs especially for pseudo-bulk eQTL analysis. We proposed a local greedy algorithm which can overcome the limitation of elbow detection algorithm and take into account the eQTL sensitivity results and the recommendations are as follows:*

*First, we calculated the percentage change of eGenes with every additional PF / PC is added. Then we performed a LOESS (locally estimated scatterplot smoothing) of the percentage change against the number of PFs / PCs (Supp Figure 7B). Instead of choosing the number of PFs / PCs, maximising the number of eGenes, we selected the number of PF just before the LOESS curve became negative. By this balanced strategy, most cell types will be only adjusted with 2~10 PFs or PCs rather than 20~50 but retain ~71% power gain of eGene discovery (Supp Figure 7C). The rationale behind this algorithm is that if the number of eGenes reaches saturation, the percentage change should be symmetrically spread around 0, so adding more factors is like adding more noise. This is consistent with the observation that most LOESS curves were randomly distributed around 0 after 10~20 factors.*

*On a different note, although adding more latent variables may lead to false discoveries in certain cell types, they can be further controlled using a permutation strategy to adjust the nominal association p-values (e.g., in fastQTL and TensorQTL software).*

2. The second and third issues pointed out on Page 2 (non-normal distribution and mean-variance dependency) also apply to bulk RNA-seq data. They should not be treated as specific to single-cell RNA-seq data.

*Reply: We acknowledge that the non-normal distribution and mean-variance relationship dependency also apply to bulk RNA-seq data. However, the degree of dependency is on a much larger scale in scRNA-seq pseudo-bulk data due to the sparsity, or more specifically, the underlying distributions of the data. For example, the expression distribution per gene in bulk RNA-seq data can be normalised using RINT method (like in GTEx study), while in scRNA-seq pseudo-bulk data, this scaling does not work well for a large proportion of genes (Supp Figure 3C-D).*

3. There are different ways to run PEER, e.g., with known covariates and without known covariates; see [1] for detail. The authors need to consider this issue. Currently the authors generate PEER factors "while including sex, age, and six genotype PCs as covariates".

*Reply: Thank you for making this point and observation. In the revised manuscript, we have addressed this by running the analyses without known covariates. We generated PEER factors without including known covariates and performed the same analyses as those for PEER factors calculated with known covariates (as in the original manuscript). The spurious correlations between PEER factors were still observed when running PEER without known covariates. We also observed no major difference in the eGene detection power under both scenarios of using all genes or HVG2000 to generate the PFs. This conclusion is consistent with the results of Zhou et al. (bioRxiv, 2022). We have added several sentences to describe the comparison results in the Methods section briefly.*

Minor comments:

1. Line 2-7, "PEER is a method that implements a Bayesian framework to estimate the latent variables and jointly learn the contribution to the gene expression variability from genotype, known factors, and hidden factors." This sentence may be edited to reflect that although the original PEER paper claims this, currently, the PEER package cannot take genotype data as an input (and known factors are optional inputs) [3].

*Reply: We have revised this sentence as suggested to be consistent with the current PEER package.*

2. Line 2-17, "the data structure will be assimilated into the bulk RNA-sequence data": do the authors mean that the data structure is similar to bulk data?

*Reply: Thank you for identifying this. We meant to say the matrix dimension of scRNA-seq pseudo-bulk data will be similar to that of the bulk RNA-seq data, which is a sample x gene matrix. We have revised the sentence to avoid confusion and ambiguity.*

3. Lines 2-17 to 2-24: the writing of this paragraph may be improved; in particular, the noun phrases may be unified to help more readers understand. From my understanding, pseudo-bulk expression data are obtained from scRNA-seq data, and bulk expression data are obtained from bulk RNA-seq data. And the main purpose of this paragraph is to introduce the differences between pseudo-bulk expression data and bulk expression data. Ideas for improvement include: in Line 19, "single-cell expression matrix" may be better replaced by "pseudo-bulk expression matrix"; in Line 20, "single-cell and bulk RNA data" may be better replaced by "pseudo-bulk expression data and bulk expression data"; in Line 23, "the underlying distribution of gene expression across cells follows either negative binomial (NB) or zero-inflated NB (ZINB) distributions" - the authors may want to clarify that this statement is regarding the pre-aggregation single cell data.

*Reply: Thanks for the suggestions to further improve this paragraph. We have highlighted the revised parts in the documents.*

*"The underlying distribution of gene expression across cells follows either negative binomial (NB) or zero-inflated NB (ZINB) distributions". This is regarding the pre-aggregation so we used “across cells” here. After pseudo-bulk aggregation, the distribution will be “across individuals”.*

4. Lines 4-3 to 4-10, "This is consistent with the observation that…": the logic of this reasoning seems unclear to me.

*Reply: Sorry for the lack of clarity here. The PEER factors capture the latent variations in the pseudo-bulk matrix. If there are few genes whose variance is much larger than others, the inferred factors that capture the variation from these genes will have much larger factor weights than other factors. However, we have reviewed this logic and reasoned that the high mean-variance dependency does not necessarily lead to this pattern (e.g., in DC and Plasma). Therefore, we removed this claim in the revised manuscript. Thank you for drawing our attention to this.*

5. Line 4-1: for the benefit of the reader, point out where these 13 options are listed in full. The Methods section doesn't seem to contain this information.

*Reply: Thank you for this suggestion. We have added a full explanation of 13 options in the Methods section in Line 13-17 Page 8.*

6. Line 4-24: what does the "skewness of genes" mean?

*Reply: Sorry for the confusion. We have changed it to “skewness of gene expression across individuals (measured by the Pearson’s moment coefficient of skewness, μ ̃_3)”. To understand how skewed the gene expression distribution is, we estimated this coefficient for all the genes and compared the median skewness coefficient for each cell type.*

*References:*

*[1] Heather J. Zhou, Lei Li, Yumei Li, Wei Li, and Jingyi Jessica Li. PCA outperforms popular hidden variable inference methods for molecular QTL mapping. bioRxiv, 2022. https://doi.org/10.1101/2022.03.09.483661.*

*[2] Anna S. E. Cuomo, Giordano Alvari, Christina B. Azodi, single-cell eQTLGen consortium, Davis J. McCarthy, and Marc Jan Bonder. Optimizing expression quantitative trait locus mapping workflows for single-cell studies. Genome Biology, 22(1):188, 2021.*

*[3] Heather J. Zhou. Capturing hidden covariates with linear factor models and other statistical methods in differential gene expression and expression quantitative trait locus studies. UCLA Electronic Theses and Dissertations, 2022. https://escholarship.org/uc/item/2rq72420.*

Reviewer #3:

Xue et. al. empirically evaluated different QC steps on pseudo-bulk matrix from single cell gene expression data for obtaining PEER factors, to deal with the issue of highly correlated PEER factors due to the violation to the assumptions of PEER methods. Sensitivity analysis was run to compare between QC and no QC. Performance on number of eGenes under different number of PEER factors is also shown for different cell types when using all genes or just the top 2000 most variable ones.

I think the methods are appropriate to the aims of the study and are well described. I just have some minor concerns:

From the methods section, we can tell that when using the top 2000 HVGs, these genes are chosen before transformation and scaling. It might be better to also make this clear somewhere in the main body of the paper. Otherwise, the ordering of the five options in line 12~18 on page 6 and the x axis labels for the last two combinations in Figure 2(A) might mislead the readers to think these top 2000 genes are chosen after the standardization.

*Reply: We thank the reviewer for their time evaluating our work and providing helpful and constructive comments.*

*We have added a sentence in Line 8-10, Page 4, to clarify that the 2000 HVGs were chosen before transformation and scaling. The same annotation is added in the legend of Figure 2.*

Are the data files available for potential readers to directly replicating the results using the provided code?

*Reply: Yes. For the OneK1K dataset, the single-cell gene expression and genotype data are available via Gene Expression Omnibus (GSE196830). The cell-by-gene data is available at Human Cell Atlas (HCA) (https://cellxgene.cziscience.com/collections/dde06e0f-ab3b- 46be-96a2-a8082383c4a1). For the fibroblast/iPSC datasets, the scRNA-seq data for all 79 fibroblast cell lines and 31 iPSC cell lines are available from ArrayExpress (Accession Number: E-MTAB-10060, https://www.ebi.ac.uk/arrayexpress/experiments/E-MTAB-10060).*

*We have added these links to the Data Availability section.*

This one is likely a typo. Line 10 on page 5, "and the gains of made in number of detected eGenes were also similar", this sentence seems not smooth. Should "of made" be deleted?

*Reply: Thanks for spotting the typo. The sentence has been corrected as “the gains of the number of detected eGenes were also similar”.*

**Second round of review**

**Reviewer 1**

My comments were well addressed.

**Reviewer 2**

I’m concerned about the key message of this paper. The main takeaway that PEER “requires additional quality control and data transformation steps on the single-cell RNA matrix to obtain valid PFs, otherwise can result in PEER factors (PFs) that are highly correlated (Pearson's correlation r = 0.626~0.997)” has already been reported in Zhou et al., 2022. Also, given the conclusion from Cuomo et al., 2021 and Zhou et al., 2022 that PCA is superior to PEER and other alternatives, this paper fails to either confirm or deny this conclusion. Further, if this paper does not invalidate the previous consensus that PCA is better, then it may not be very meaningful to discuss the QC and data transformation required for using PEER. One idea for the authors to consider: for each QC/data transformation method, compare the number of discoveries made using PEER factors (where the number of PEER factors is chosen using the authors’ “local greedy” method) and the number of discoveries made using PCs (where the number of PCs is chosen via BE, for example). The result from this analysis, if done, should be put in the main text rather than the supplementary.

I have a few additional comments:

1. The result in Figure S10 seems problematic because nonzero PCs are guaranteed to have exactly zero sample covariance and therefore exactly zero sample correlation (Zhou et al., 2022). Therefore, the small, non-zero mean pair-wise correlations in (a) are merely numerical inaccuracies, and the high max pair-wise correlations in (b) seem wrong.

2. In Figure S7, how is “relevant variance explained by each factor” for PEER factors defined? From my understanding, relevant variance explained may not be a valid concept for PEER because it does not consider the weights assigned to each PEER factor for each gene in the estimated PEER model. In other words, I don’t think the automatic elbow detection method in PCAForQTL is applicable to PEER, contrary to the authors’ belief (Line 5-19).

3. In Line 1-17, the authors wrote “However, its performance has not been evaluated in single-cell eQTL data analysis”. This is not true given Cuomo et al., 2021.

4. Zhou et al. bioRxiv, 2022 is now published: https://doi.org/10.1186/s13059-022-02761-4. Please update the reference.

**Authors Response**

**Point-by-point responses to the reviewers’ comments:**

Reviewer #1:

My comments were well addressed.

*Reply: We thank you again for the comments, which helped us improve the manuscript.*

Reviewer #2:

I’m concerned about the key message of this paper. The main takeaway that PEER “requires additional quality control and data transformation steps on the single-cell RNA matrix to obtain valid PFs, otherwise can result in PEER factors (PFs) that are highly correlated (Pearson's correlation r = 0.626~0.997)” has already been reported in Zhou et al., 2022. Also, given the conclusion from Cuomo et al., 2021 and Zhou et al., 2022 that PCA is superior to PEER and other alternatives, this paper fails to either confirm or deny this conclusion. Further, if this paper does not invalidate the previous consensus that PCA is better, then it may not be very meaningful to discuss the QC and data transformation required for using PEER. One idea for the authors to consider: for each QC/data transformation method, compare the number of discoveries made using PEER factors (where the number of PEER factors is chosen using the authors’ “local greedy” method) and the number of discoveries made using PCs (where the number of PCs is chosen via BE, for example). The result from this analysis, if done, should be put in the main text rather than the supplementary.

*Reply: We thank the reviewer for highlighting this discussion on a key message of our paper.*

*We agree with the conclusions from Zhou et al. 2022 and acknowledge that in our previous revision, we did not draw a confirmatory conclusion on this point. We have rectified this now. We also agree that PCA is easier to implement and faster than PEER. Regarding eGene discovery, our comparison (Figure S13) shows that the PCA identified very similar numbers of eGenes compared to PEER, with slight improvements in performance for PCA for almost all cell types. These results align with the conclusion Zhou et al. 2022 (their Section 2.3), and we have amended our test to support this.*

*We feel it is important to include results showing variable performance over different QC methods. In our experience, depending on the experimental design, there are often specific reasons (unrelated to the eQTL analysis) for using certain QC steps in single-cell data. As such, we think it is important to demonstrate the impact of calculating the latent variables and, subsequently, eQTL detection power. This is also something that was not discussed in Zhou et al. and Cuomo et al. papers, which focused on bulk RNA and one scRNA-seq QC approach, in one cell type respectively.*

*Thank you for the suggestion on the comparing the number of discoveries used PEER (where the number of factors is chosen using the ‘local greedy’ method, vs PCs (where the number of PCs is chosen using BE). We have completed this comparison and included the results in the main text. We first ran the BE algorithm for PCs inferred from the single cell data for each QC/data transformation options. The results show that the BE algorithm was selecting a large number of PCs (100-200), while automatic elbow detection method selected mostly 3~22 (Figure 2, Figure S7). We speculate that this is because in the single cell data, the first a few PCs explain the most variation. In the BE algorithm, randomly selecting K PCs will very likely choose the PCs explained a tiny proportion of variance. So, when comparing variance explained by the first K PCs and randomly selected K PCs, the former is often larger.*

*We further compare the elbow detection method and local greedy algorithm for PCA (Figure S9). The elbow detection method identified on average 0.6% less eGenes than the local greedy algorithm but the optimal number of PCs was much higher (31 vs 7) across 14 cell types. Notably, in CD4_SOX4 cells, the elbow detection method identified 20.5% less eGenes than the local greedy algorithm, which shows its disadvantage that it did not take sensitivity results into account. We thank the reviewer for this suggestion which adds value to the paper. We have added the new results in the main text.*

*Altogether, one of the key messages of this paper is that inferring latent variables is different between bulk RNA-seq and single-cell RNA-seq data. Our QC/ data transformation methods and local greedy algorithm specifically designed for single cell data, and the results show that such methods can also benefit the inference using PCA in single cell data.*

I have a few additional comments:

1. The result in Figure S10 seems problematic because nonzero PCs are guaranteed to have exactly zero sample covariance and therefore exactly zero sample correlation (Zhou et al., 2022). Therefore, the small, non-zero mean pair-wise correlations in (a) are merely numerical inaccuracies, and the high max pair-wise correlations in (b) seem wrong.

*Reply: Thank you for flagging this. We’ve looked into this and observed that the high pair-wise correlation between PCs is mainly because the QC options 1-5 did not centre and scale each feature. In the Github page of Zhou et al., they “suggest always centering and scaling to be safe”. We double checked that the R function of PCA, prcomp sets the “center” and “scale” as default but in our case, we want to test the extreme case so we manually turned off these two flags when generating PCs.*

*We confirm that if we QC options that centre and/or scale the data (options 6-14), all the correlations are zeros (empirically the correlation estimates are 1.6E-15 ~ 4.5E-14 on the computer). We have added this note in the legend of Figure S10 (now Figure S12).*

2. In Figure S7, how is “relevant variance explained by each factor” for PEER factors defined? From my understanding, relevant variance explained may not be a valid concept for PEER because it does not consider the weights assigned to each PEER factor for each gene in the estimated PEER model. In other words, I don’t think the automatic elbow detection method in PCAForQTL is applicable to PEER, contrary to the authors’ belief (Line 5-19).

*Reply: We thank the review for pointing out the conceptual difference. The “relevant variance explained by each factor” for PEER is defined as factor relevance (or inverse of the ARD parameters) in the PEER protocol paper (Stegle et al. Nature Protocol. 2012), where ARD stands for “automatic relevance determination” indicating the uncertainty of the factor. We agree with the reviewer’s concern that the elbow detection method may not be directly applicable for PEER as this relevance factor is different from explained variance for each PC, even though the plot also has a natural elbow (the authors of the PEER mentioned this in their demo code at https://github.com/PMBio/peer/blob/master/examples/r_demo.R).*

*We have followed the suggestion from the reviewer and only compare the PEER + “local greedy” algorithm and PCA + Elbow detection method (already showed that PCA + BE is not comparable as mentioned above). The results are updated in Figure S7-9. The main conclusion did not change for this comparison because the inferred PFs and PCs using option 12 are similar (Figure S13).*

3. In Line 1-17, the authors wrote “However, its performance has not been evaluated in single-cell eQTL data analysis”. This is not true given Cuomo et al., 2021.

*Reply: Thanks for pointing out the inaccurate claim and we apologize that a “largely” was accidently deleted during the resubmission. We re-written this sentence and revised it as “However, their performance has not been evaluated extensively in single-cell eQTL data analysis, especially for different cell types or quality control methods”.*

*Our evaluation provides more features of how PEER factors perform in single-cell eQTL data analysis than Cuomo et al. 2021, given*

*• We tested maximum 50 factors while Cuomo et al. tested 25;*

*• We fitted the inferred factors incrementally by 1 at a time in the sensitivity test, while Cuomo et al. chose 5;*

*• We evaluated the performance in 14 cell types while Cuomo et al. evaluated induced pluripotent stem cell (iPSC) only.*

4. Zhou et al. bioRxiv, 2022 is now published: https://doi.org/10.1186/s13059-022-02761-4. Please update the reference.

*Reply: We have updated the reference and citation throughout the manuscript.*

**Third round of review**

**Reviewer 2**

I hope the authors can improve the content organization and writing for better clarity. I don't have other comments.

**Authors Response**

**Point-by-point responses to the reviewers’ comments:**

Reviewer #2:

I hope the authors can improve the content organization and writing for better clarity. I don't have other comments.

*Reply: We reformatted the paper slightly to help improve the layout and readability.*
